# Long Chain Fatty Acids as Modulators of Immune Cells Function: Contribution of FFA1 and FFA4 Receptors

**DOI:** 10.3389/fphys.2021.668330

**Published:** 2021-07-01

**Authors:** Maria A. Hidalgo, Maria D. Carretta, Rafael A. Burgos

**Affiliations:** Laboratory of Inflammation Pharmacology, Institute of Pharmacology and Morphophysiology, Universidad Austral de Chile, Valdivia, Chile

**Keywords:** fatty acids, immune, FFA1, FFA4, T-cells, neutrophils, macrophages

## Abstract

Long-chain fatty acids are molecules that act as metabolic intermediates and constituents of membranes; however, their novel role as signaling molecules in immune function has also been demonstrated. The presence of free fatty acid (FFA) receptors on immune cells has contributed to the understanding of this new role of long-chain fatty acids (LCFAs) in immune function, showing their role as anti-inflammatory or pro-inflammatory molecules and elucidating their intracellular mechanisms. The FFA1 and FFA4 receptors, also known as GPR40 and GPR120, respectively, have been described in macrophages and neutrophils, two key cells mediating innate immune response. Ligands of the FFA1 and FFA4 receptors induce the release of a myriad of cytokines through well-defined intracellular signaling pathways. In this review, we discuss the cellular responses and intracellular mechanisms activated by LCFAs, such as oleic acid, linoleic acid, palmitic acid, docosahexaenoic acid (DHA), and eicosapentaenoic acid (EPA), in T-cells, macrophages, and neutrophils, as well as the role of the FFA1 and FFA4 receptors in immune cells.

## Introduction

The interplay between metabolism and immunity has been described by several authors. Fatty acids play an essential role in metabolic disorders and in chronic diseases wherein inflammation is associated. Several pieces of evidence suggest a relationship between obesity, in which there is an increase in levels of fatty acids, and impaired immune response (Falagas and Kompoti, 2006; Boden, 2008; Sheridan et al., 2012).

The physiological functions of fatty acids on immune cells are similar to those of non-immune cells, such as energy source and membrane constituents, but they also regulate immune homeostasis through anti-inflammatory or pro-inflammatory effects. Furthermore, new roles as signaling molecules have begun to be described (de Jong et al., 2014; Im, 2016; Rodrigues et al., 2016). Those functions on immune response are diverse and mainly dependent on the type of fatty acid and immune cell (Kimura et al., 2020). For instance, omega-3 fatty acids have recognized anti-inflammatory effects as having been demonstrated in T-cells and inflammatory cells, such as macrophages and neutrophils. On the contrary, saturated fatty acids have pro-inflammatory effects.

Long-chain fatty acids are saturated or unsaturated fatty acids containing 13–21 carbons. Oleic, linoleic, palmitic, docosahexaenoic acid (DHA), and eicosapentaenoic acid (EPA) are important in health and disease. Levels of some fatty acids (oleic acid, palmitic acid, or linoleic acid) are higher in the blood compared with other fatty acids, and their levels can increase significantly in diabetes mellitus, insulin resistance, and obesity (Prescott et al., 1999; Boden, 2008). Linoleic acid is an essential fatty acid and the most abundant type of polyunsaturated fatty acid (PUFA) in the human diet. Some fatty acids (DHA and EPA) are crucial in mediating inflammation. It is well-known that a diet with high content of monounsaturated fatty acids, specifically oleic acid, exerts a beneficial effect reducing the risk of coronary heart diseases (Kris-Etherton, 1999). Likewise, the role of fatty acids in some diseases, such as cancer, inflammation, and autoimmune diseases, has been well-discussed in several reviews (Simopoulos, 2008; Rohrig and Schulze, 2016; Innes and Calder, 2018; Li et al., 2019). Oleic and linoleic fatty acids, also known as omega-9 and−6 fatty acids, respectively, are monounsaturated fatty acids with plasma total lipid levels ranging from 0.03 to 5 mmol/L (0.03–3.2 mmol/L for oleic acid, and 0.2–5 mmol/L for linoleic acid). DHA and EPA are polyunsaturated fatty acids that are also known as omega-3 fatty acids, ranging between 7.2 and 237.5 μmol/L for DHA (Abdelmagid et al., 2015). On the contrary, palmitic acid is a saturated fatty acid with plasma total lipid levels ranging from 0.3 to 4.1 mmol/L (Abdelmagid et al., 2015).

Long-chain fatty acids exert their actions in part through membrane receptors, which were first described in the pancreas and intestinal cells (Briscoe et al., 2003; Itoh et al., 2003; Milligan et al., 2017). However, these receptors have been detected in immune cells, such as macrophages and neutrophils (Oh et al., 2010; Manosalva et al., 2015; Agrawal et al., 2017; Souza et al., 2020). The purpose of this review is to discuss the role of long-chain fatty acids (LCFAs) and their receptors in key cells that are part of the innate and adaptive immune response. Here, we focus on the effect of LCFAs on immune cells, specifically T-cells, neutrophils, and macrophages, and the intracellular signaling pathways activated by LCFAs in these cells. Also, the presence and role of free fatty acids (FFAs) receptors in immune cells are discussed.

## Effects of LCFA on Immune Cells

Neutrophils and macrophages are considered the first line of defense of the host against injury or pathogen invasion, as part of the innate immunity, while T-cells are the main constituent of the adaptive immune response. However, the current evidence recognizes an interplay between innate and adaptive immunity (Gregersen and Behrens, 2006; Mantovani et al., 2011). We will discuss the effects of LCFA on T-cells, neutrophils, and macrophages because of their important role in the orchestration of immune response.

### Oleic Acid

The effect of oleic acid on T-cells has not yet been extensively studied. However, existing studies have suggested a positive effect on T-cells through different mechanisms. Oleic acid increased the proliferation of human lymphocytes isolated from peripheral blood (Gorjao et al., 2007; Ioan-Facsinay et al., 2013) and reduced the proliferation of Jurkat T-cells (Verlengia et al., 2003). Two recent studies have shown mechanisms by which oleic acid suppressed T-cell function. Oleic acid has been shown to enhance human Treg suppressive function through a mechanism that involves amplification of Treg fatty acid oxidation-driven oxidative phosphorylation metabolism, resulting in positive feedback that increases the expression of FOXP3 and phosphorylation of STAT5 (Pompura et al., 2020). Fatty acids can also alter cell function by inducing changes in epigenetic processes (Burdge and Lillycrop, 2014; Silva-Martinez et al., 2016). Oleic acid at physiologically relevant concentrations induced CpG locus-specific DNA methylation associated with zinc-finger-containing transcription factors in Jurkat T cells (Perez-Mojica et al., 2020). These findings are significant since epigenetic changes have been linked with diverse diseases, such as asthma, systemic lupus erythematosus, rheumatoid arthritis, multiple sclerosis, and diabetes mellitus (Zhang et al., 2020). Therefore, the unveiling of this mechanism opens new opportunities to develop a dietary intervention based on a combination of fatty acids for immune-metabolic diseases.

Studies on the effect of oleic acid on neutrophil function show two types of response, depending on whether oleic acid is studied alone, or in the presence of a stimulus. In *in vivo* assays, oleic acid reduced the migration and rolling of neutrophils when administered by gavage in a mouse model of sepsis (Medeiros-de-Moraes et al., 2018). Similarly, in isolated human neutrophils, oleic acid reduced lipopolysaccharide (LPS)-induced cell migration (Reyes-Quiroz et al., 2014). In a study on wound healing in rats, oleic acid topically administered showed beneficial effects on the inflammatory phase. In this model, an increase in the number of neutrophils in the wounded area and air pouches was observed, which can speed up wound healing (Pereira et al., 2008). Most studies on neutrophils in the absence of a stimulus have described a stimulatory effect of oleic acid. Oleic acid increases reactive oxygen species (ROS) production in humans, rats, and bovine neutrophils in *in vitro* assays (Hatanaka et al., 2006; Carrillo et al., 2011; Hidalgo et al., 2011). It also increased the release of vascular endothelial growth factor-alpha and interleukin-1beta (IL-1β) in rat neutrophils (Pereira et al., 2008). In bovine neutrophils, oleic acid increased several effects or functions, such as the release of matrix metalloproteinase-9 (MMP-9), IL-8, and cyclooxygenase-2 expression (Hidalgo et al., 2011; Manosalva et al., 2015; Mena et al., 2016). At high concentration, oleic acid induced the release of ATP and neutrophil extracellular traps (NETs), which is mediated *via* pannexin-1 dependent ATP release (Alarcon et al., 2020). Although oleic acid was used at high concentrations in these assays, its results are relevant in postpartum cow, because they show high plasma levels of oleic acid in this period. Overall, all the evidence of the effects of oleic acid on neutrophil function shows that this fatty acid could be useful for the destruction of microorganisms or during tissue injury.

The effect of oleic acid on macrophages has been determined in both *in vivo* and *in vitro* studies, and overall they have shown an anti-inflammatory effect. *In vivo* and *in vitro* experiments suggest that dietary oleic acid can increase M2 macrophage polarization. Camell and Smith (2013) observed that a diet high in oleic acid elevated the levels of M2 macrophages in the adipose tissue of mice and increased the levels of M2 macrophage markers (CD206, MGL1, and ARG1) in RAW264.6 macrophages under *in vitro* conditions. In another line of evidence, oleic acid induced the secretion of apolipoprotein E (apoE) *via* a post-translational glycosylation to increase its secretion in monocyte-derived macrophages, which play a key role in metabolic disorders and atherosclerosis (Huang et al., 2004). In addition, oleic acid decreased the LPS-induced expression of iNOS, COX-2, TNF-α, IL1β, and IL6 mRNAs in murine RAW264.7 macrophages, and reduced nitric oxide formation and palmitate-induced apoptosis (Kim et al., 2017). Furthermore, oleic acid inhibited saturated fatty acid-induced IL-1β release in a dose-dependent manner in murine primary macrophages (Karasawa et al., 2018). In summary, all the effects of oleic acid suggest that it is involved in mediating anti-inflammatory responses with immune cells.

Interestingly, products of the nitration of fatty acids have shown potent anti-inflammatory properties, and some studies show their action on immune cells. Nitro-oleic acid induced IL-10 expression and accumulation of Treg cells in a model of hypersensitivity in mice (Mathers et al., 2017). Also, some actions on neutrophils have been described; nitro-oleic acid induced apoptosis of neutrophils and their phagocytosis by alveolar macrophages in a model of allergic airway disease (Reddy et al., 2013) and reduced neutrophil infiltration in a murine model of myocardial ischemia and reperfusion (Rudolph et al., 2010). Nitro-oleic acid reduced the activation of signaling pathways, such as STAT5, ERK, and PI3K, and the expression of adhesion molecules and pro-inflammatory cytokines in RAW 264.7 macrophages or mice bone marrow-derived macrophages (Rudolph et al., 2010; Ambrozova et al., 2016; Verescakova et al., 2017). Furthermore, nitro-oleic acid (5 μM) increased the expression of CD36 in RAW 264.7 macrophages, and ligand binding analysis showed the interaction of nitro-oleic acid with CD36 (Vazquez et al., 2020). This effect of nitro-oleic acid could be dependent on the cellular model and concentrations used, because Lamas Bervejillo (Lamas Bervejillo et al., 2020), using THP-1 cells differentiated to macrophages and 1 μM nitro-oleic acid, did not observe changes in CD36 expression. These different results show potential limitations of some *in vitro* studies, depending on the experimental setting of each assay.

### Linoleic Acid

The immunoregulatory effects of fatty acids have also been observed with linoleic acid, and they are especially interesting in patients who consume high-fat diets. Several years ago, some *in vivo* studies began to show the role of fatty acids in the immune response using dietary fatty acids in rats fed with high-fat diets, and also through *in vitro* assays (Jeffery et al., 1996, 1997). These studies observed a stimulatory effect of linoleic acid on T-cells proliferation. Then, most recent evidence suggests that linoleic acid might decrease the function of immune cells. Huang et al. (2020) demonstrated that linoleic acid inhibited Th1 and Th17 cell differentiation and reduced IL-17 and IFN-γ production, using isolated human and mouse CD4^+^T-cells. Likewise, linoleic acid induced death in CD4^+^T-cells and promoted apoptosis and necrosis in Jurkat T cells (Cury-Boaventura et al., 2004; Ma et al., 2016). Although existing studies show a suppressive effect of linoleic acid on T cell activation, more studies are needed to demonstrate the mechanisms involved in these effects.

Studies on neutrophils suggest the participation of linoleic acid, through its metabolite 13-S-hydroxyoctadecadienoic acid (HODE), in airway inflammation. The *in vivo* study of Mabalirajan et al. (2013) observed that mice treated with 13-S-HODE showed airway neutrophilia. Then, Panda et al. (2017) showed that mice with allergic airway inflammation treated with dexamethasone and 13-S-HODE increased neutrophilic inflammation. These findings are very important for obese patients, because they suggest that the metabolism of linoleic acid could affect asthma severity and sensitivity to steroids. In addition to these *in vivo* pro-inflammatory effects, *in vitro* studies have shown that linoleic acid increases several neutrophil responses, such as oxidative stress, production of thromboxane, TNF-α, COX-2, IL-8, IL-1β and CINC-2αβ, MMP-9 release, ROS, and NET release (Vaughan and Walsh, 2005; Hidalgo et al., 2011; Manosalva et al., 2015; Mena et al., 2016; Alarcon et al., 2020). A pro-inflammatory stage is crucial for the inflammatory phase in wound healing, and linoleic acid has shown to be useful in this process. Similar to the aforementioned effects of oleic acid on neutrophils described by Pereira et al. (2008), linoleic acid also increased the release of vascular endothelial growth factor-alpha and IL-1β in neutrophils during the wound healing process, which can speed up wound healing.

Linoleic acid also exerts pro-stimulatory effects on macrophages; however, depending on the experimental design of the studies, an anti-inflammatory effect has also been suggested. Linoleic acid increased the mRNA expression of pro-inflammatory markers TNF-α, IL-6, chemokine (C-C motif) ligand 2 (Ccl2), and IL-1β in mouse peritoneal macrophages (Kain and Halade, 2019). Also, linoleic acid increased the ROS production in macrophages-derived THP-1 cells and reduced the mRNA corresponding to the antioxidant enzymes catalase, glutathione peroxidase, and superoxide dismutase-1; however, it did not affect their enzymatic activities (Rybicka et al., 2011). In contrast, an anti-inflammatory effect of linoleic acid was suggested by Magdalon et al. (2012). The authors observed a reduction in the levels of IL-1β and IL-6 in peritoneal macrophages isolated from rats after oral administration of linoleic acid; however, they also observed an increase in IL-1β and decrease in levels of IL-10 in LPS-treated macrophages, which suggests that it may lead to higher microbicidal activity in these cells. Besides, linoleic acid might play a protective role against parasitic infection. Linoleic acid suppressed the parasitic load of *Leishmania donovani* in J774A.1 macrophages and promoted a Th-1-mediated immune response (Saini and Rai, 2020).

### Palmitic Acid

Palmitic acid is a fatty acid known to stimulate pro-inflammatory responses and is of special interest in conditions, such as obesity, type 2 diabetes or patients that consume high-fat diets. Although palmitic acid is the most abundant saturated fatty acid in plasma, its effects on T cells and neutrophils have been poorly studied. On the contrary, more antecedents exist describing the effect of palmitic acid on macrophages.

Several years ago, some studies suggested that palmitic acid can modulate the T-cell function. An *in vivo* study conducted on rats fed with a diet rich in palmitic acid showed increased lymphocyte proliferation compared with rats fed with diets rich in other fatty acids (oleic acid, linoleic acid, or fish oil). However, this study also analyzed the levels of plasma lipids. According to these results, the authors suggested that the ratio of different dietary fatty acids could be more relevant than the individual amount of each fatty acid (Tinahones et al., 2004). Besides, it was shown that palmitic acid regulates DNA synthesis (low concentrations of palmitic acid stimulated DNA synthesis, whereas high concentrations inhibited it) and enhances cytokine release (IFN-γ, TNF-α, IL-6, IL-8, IL-1β, IL-2, and IL-10) in human lymphocytes (Karsten et al., 1994; Stentz and Kitabchi, 2006). Other studies demonstrated that low concentrations of palmitic acid induced proliferation and that high concentrations were cytotoxic in Jurkat cells and human lymphocytes (Lima et al., 2002; Gorjao et al., 2007; Takahashi et al., 2012). Although the exact mechanisms on how palmitic acid increases those responses in T-cells are not understood, more recent reports have attempted to clarify them. As mentioned above, palmitic acid plays a detrimental role in patients who are obese or have type 2 diabetes, where its levels in plasma are elevated. The study of Zhou et al. (2019) showed that the treatment of human T-cells with palmitic acid led to significant upregulation of signaling lymphocytic activation molecule family member 3 (SLAMF3) in cells primed with anti-CD3/CD28 antibodies. This finding is significant to understand the mechanisms by which palmitic acid promotes inflammation in patients with type 2 diabetes, which show the upregulation of SLAMF3 on T cells compared with healthy persons, and SLAMF3 has been associated with increased production of pro-inflammatory cytokines. Another recent study has shown evidence about the role of a subset of CD3+ T cells in fatty liver diseases, such as steatohepatitis, in which chronic accumulation of free fatty acids and activated inflammatory response are observed. The authors showed that the subset of mouse CD3+ T cells stimulated by palmitic acid induces the expression of IL17A (Torres-Hernandez et al., 2020), which has been implicated in recruiting immune cells during the early stages of liver disease.

In neutrophils, evidence from several years ago has shown somewhat contradictory results. Palmitic acid decreased hydrogen peroxide generation in cells stimulated with zymosan, without affecting other neutrophil functions (chemotaxis, phagocytosis, superoxide radical anion, and hydroxyl radical generation) (Akamatsu et al., 2001). In contrast, Wanten et al. (2002) observed that high concentrations of palmitic acid increased ROS production in the absence of any stimulus. The authors attributed these differences to the distinct methods used in the studies. In a more recent study, the effect of palmitic acid on neutrophils has also been assessed to evaluate its contribution to the local inflammation in obesity. Exposure of murine macrophages to palmitic acid induces the release of nucleotides through pannexin-1 channels that attract neutrophils, suggesting that this mechanism may facilitate the recruitment of neutrophils into metabolic tissues during obesity (Tam et al., 2020).

Numerous studies exist detailing the pro-inflammatory effects of palmitic acid on macrophages (Kim et al., 2017; Wang et al., 2017; Karasawa et al., 2018; Korbecki and Bajdak-Rusinek, 2019; Sano et al., 2020; Youk et al., 2021). High-fat diet (HFD) may promote hepatocarcinogenesis through an exacerbated inflammatory response that affects the liver function. *In vivo* experiments demonstrated that HFD affected liver function in mice, induced liver infiltration of macrophages, and increased expression of inflammatory factors, such as proliferating cell nuclear antigen (PCNA), NF-κB, cyclin D1, TNF-α, and IL-1β in the liver (Fu et al., 2020). In addition, to understand the link between obesity and inflammation, and its effects in pregnancy, Sano et al. (2020) studied the *in vivo* and *in vitro* effects of palmitic acid in pregnant mice. The authors observed that palmitic acid induced ASC assembly and IL-1β release in murine macrophages. Palmitic acid-induced IL-1β release was suppressed in NLRP3-knockdown macrophages, which, together with *in vivo* experiments, suggests that higher levels of palmitic acid exposure in pregnant mice activate NLRP3 inflammasome and induce placental inflammation, which may result in pregnancy complications (Sano et al., 2020). Also, an *in vitro* assay showed that palmitic acid enhanced macrophage adhesion and decreased phagocytosis, as well as increased nitric oxide production by J774 cells (Calder et al., 1990a; de Lima et al., 2006). Recent evidence intends to explain the role of palmitic acid in inflammatory responses in obesity and insulin resistance (Korbecki and Bajdak-Rusinek, 2019). Palmitic acid elevated the release of IL-1β, IL-6, and TNF-α, which are important cytokines that cause insulin resistance, increase apoptosis, and activate the NLRP3 inflammasome in macrophages (Kim et al., 2017; Wang et al., 2017; Karasawa et al., 2018; Korbecki and Bajdak-Rusinek, 2019; Sano et al., 2020; Youk et al., 2021). Also, palmitic acid induced M1 macrophage polarization and increased CD36 expression, a membrane glycoprotein that transports fatty acids, *via* decreased expression of the histone methyltransferase G9a (Lu et al., 2017; Wang et al., 2021). An *in vitro* assay demonstrated that palmitic acid induced the expression of iNOS and secretion of IL-6, IL-10, CCL2, IFN-γ, and TNF-α in mouse primary macrophages (Fu et al., 2020). Thus, the most recent studies have elucidated the role of palmitic acid not only in mediating the function of isolated macrophages but also the pro-inflammatory effects on macrophages in conditions such as obesity or when HFDs are ingested.

### Omega-3 Fatty Acids: Docosahexaenoic Acid and Eicosapentaenoic Acid

Numerous beneficial effects of omega-3 fatty acids have been proposed, and their mechanisms of action have begun to be elucidated. DHA and EPA, the main dietary n-3 fatty acids present in fatty fish, have shown positive effects on health and diseases, such as cardiovascular diseases, dyslipidemia, hypertension, atherosclerosis, diabetes, obesity, gastrointestinal disorders, arthritis, and neurological disorders (reviewed in Riediger et al., 2009; Yashodhara et al., 2009). Omega-3 fatty acids not only participate in the formation of specialized pro-resolving mediators (SPMs) of the D- or E-series (Jaudszus et al., 2013; Serhan, 2014), they also participate in the orchestration of the anti-inflammatory response by inhibiting the expression of pro-inflammatory factors and preventing the hyper-immune response (Riediger et al., 2009; Yashodhara et al., 2009; Rodrigues et al., 2016). A mechanism that could in part explain the effect anti-inflammatory of n-3 fatty acids is its participation in the synthesis of 3-series of prostaglandins (*via* competition with arachidonic acid by the COX pathway) and reduction of the levels of 2-series of prostaglandins (i.e., it has been demonstrated that PGE3 reduces the level of IL-6 compared with PGE2 in RAW264.7 macrophages) (Bagga et al., 2003). Two other mechanisms had been proposed prior to the discovery of DHA as a ligand of the FFA4 receptor, the activation of peroxisome proliferation activator receptor gamma (PPARγ), and the inhibition of TLR2/3/4 (Lee et al., 2003; Gani and Sylte, 2008). However, with the study conducted by Oh et al. (2010) examining the anti-inflammatory mechanism through the FFA4 receptor, several effects of n-3 fatty acids were elucidated.

The first studies that evaluated the effects of n-3 fatty acids on the immune system were performed using diets containing fatty fish or diets enriched with DHA or EPA, and they suggested a beneficial effect (Meydani et al., 1991; Fowler et al., 1993; Peterson et al., 1998; Fritsche et al., 1999; Arrington et al., 2001a,b). Several studies have suggested the usefulness of omega-3 fatty acids in the managing of diseases associated with an exacerbated immune response. Oral administration of DHA and EPA to asthmatic patients for 3 months showed a reduction in the serum levels of IL-17A and TNF-α compared with those observed after the administration of placebo, suggesting that these fatty acids are a promising complementary approach for managing asthma (Farjadian et al., 2016). The effect of EPA on experimental autoimmune encephalomyelitis (EAE) in mice was also assessed; the authors described a reduction in the production of IFN-γ and IL-17 and an increase in the expression of peroxisome proliferator-activated receptors in CD4 T cells infiltrating the central nervous system (Unoda et al., 2013). Also, DHA and EPA reduced the migration of T cells *in vivo* and *in vitro* (Yessoufou et al., 2009; Cucchi et al., 2020). Other studies have evaluated the effect of purified n-3 fatty acids; DHA or EPA showed an anti-proliferative effect, reduced the expression of IL-2, IFN-γ, and IL-10 in Jurkat T-cells, and induced hypermethylation in DNA (Verlengia et al., 2004; Perez-Mojica et al., 2020; Saidi et al., 2020). DHA could be useful in anticancer chemotherapy. The treatment of Jurkat T-cells with DHA and the anticancer drugs everolimus and barasertib induced apoptosis and ROS production; however, these effects were not observed in normal lymphocytes (Zhelev et al., 2016). Controversial data about the beneficial effect of n-3 fatty acids in cancer have been demonstrated by some clinical studies with omega-3 supplementation in patients with cancer. However, these discrepancies could be attributed to difficulties in collecting dietary data or genetic variations affecting PUFA metabolism (reviewed in Azrad et al., 2013).

Similar as in T cells, some studies have assessed the effects of dietary supplementation with DHA on neutrophil functions; however, the effects on oxidative response are somewhat divergent. Capo et al. (2015) observed that DHA diet supplementation increased the response (antioxidant and anti-inflammatory) of neutrophils to *in vitro* phorbol myristate acetate (PMA) activation, whereas Gorjao et al. (2006) observed an increase in ROS production by PMA-stimulated neutrophils, in addition to an increase in the phagocytic activity of zymosan particles. Neutrophils play a crucial role in the inflammatory phase of a healing wound. The oral administration of EPA and DHA to patients with chronic venous leg ulcers reduced the number of CD66b, an activated neutrophil marker, and the area of the wound, suggesting that EPA + DHA therapy could regulate PMN activity and stimulate healing (McDaniel et al., 2017). In the same way, dietary administration of EPA and DHA to mice with contact dermatitis in the ear reduced ear inflammation and neutrophil infiltration, with EPA being more efficient (Sierra et al., 2008). DHA and EPA also induce different *in vitro* responses in neutrophils, such as the production of hydrogen peroxide, phagocytic capacity, fungicidal activity, TNF-α, IL-1β, and MMP-9 granule release, and ROS production in rat, goat, or bovine neutrophils (Pisani et al., 2009; Paschoal et al., 2013; Olmo et al., 2019). DHA reduces the IL-8 release induced by pneumolysin in human neutrophils and PMA-induced ROS production in goat neutrophils (Fickl et al., 2005; Pisani et al., 2009).

In macrophages, diverse studies performed under *in vivo* conditions have demonstrated a beneficial effect of DHA and the role of macrophages in cancer, type-1 diabetes, and diabetic wound healing (Jia et al., 2020; Liang et al., 2020; Davanso et al., 2021). Dietary n-3 fatty acids inhibited M2-like macrophage function and the development and progression of castrate-resistant prostate cancer in an immunocompetent mouse model (Liang et al., 2020). Furthermore, dietary DHA significantly accelerated wound healing in diabetic rats by restoring the impaired plasticity of macrophage progenitor cells (Jia et al., 2020). Another study suggested the use of DHA as adjuvant therapy in the treatment of type-1 diabetes. The authors observed that DHA reduced the macrophage inflammatory state and reversed diabetes-induced changes (Davanso et al., 2021). A diet containing DHA and EPA showed anti-inflammatory effects and reduced atherogenesis in Apoe–/– mice (Takashima et al., 2016). Also, a HFD supplemented with EPA administrated to mice reduced macrophage infiltration, adipose tissue inflammation, and adipocyte hypertrophy (LeMieux et al., 2015). Evidence *in vitro* has also shown diverse effects of DHA on macrophages (Im, 2016); DHA reduced the expression of histone deacetylase 3, 4, and 5 in RAW 264.7 macrophages, which could affect the posttranslational modifications of histones, resulting in the alteration of gene expression, thus explaining the possible anti-inflammatory mechanism of DHA (Pham et al., 2018). DHA also reduced crystalline silica-induced inflammasome activation and IL-1 release in macrophages (Wierenga et al., 2019). Kawano et al. (2019) showed that DHA induced the expression of M2 macrophage markers, suggesting that it controls the M2 macrophage polarization that plays a key role in innate immunity. Thus, the results of diverse studies are consistent with respect to the anti-inflammatory effect of omega-3 fatty acids on immune cells.

T-cells, neutrophils, and macrophages are key cells in the orchestration of the immune response, as mentioned above. However, some effects of LCFAs on other immune cells, such as B-cells and dendritic cells (DCs), have been described although to a lesser extent. Among the effects described in B-cells, an increase in radical oxygen species release and cell proliferation have been observed (Butler et al., 1997; Campoio et al., 2011). However, other authors suggest that a mix of LCFAs, in the range of 0.1–0.4 mM, induces apoptosis of B-cells (Otton and Curi, 2005). Moreover, an individual LCFA can reduce the proliferation of B-cells induced by LPS (Calder et al., 1990b). This discrepancy could be attributed to the concentrations of the LCFA, or because the potency of the LCFA is determined by the fatty acid: BSA ratio, or the simultaneous addition of a saturated and an unsaturated fatty acid abrogates the inhibitory activity of the former (Buttke, 1984). Palmitic and oleic acids sensitize DCs resulting in augmented secretion of TH1/TH17-instructive cytokines upon pro-inflammatory stimulation (Stelzner et al., 2016). DHA induces the upregulation of activation markers on DCs (Carlsson et al., 2015). LCFAs and, more specifically, adipose-derived fatty acids stimulate hepatic DCs accumulation, suggesting a key role in the pathology of the obese liver (Harmon et al., 2018).

## Intracellular Signaling Pathways Activated by Long-Chain Fatty Acids in Immune Cells

Although several evidence has described the effects of LCFAs on immune cells, the current information on the intracellular signaling pathways activated by LCFAs in immune cells is still reduced. In contrast, most studies have described the effects and intracellular signaling activated by LCFAs in other cell types, such as pancreatic and intestinal cells, because of their relevance in metabolic diseases. However, those cells are not the focus of this review and, therefore, will not be reviewed here (a review can be found in Kimura et al., 2020).

### Oleic Acid

Few studies have been published examining the effects of oleic acid on T-cells, and even fewer studies have evaluated the signaling pathways induced by this fatty acid in T-cells. A recent assay has shown the effects of oleic acid on intracellular calcium, a crucial second messenger involved in processes such as proliferation and IL-2 expression through the pathway calcineurin/NFAT. The authors have shown that oleic acid increased intracellular calcium, partly mediated by extracellular calcium influx through econazole-insensitive channels (Carrillo et al., 2017). They have also observed that oleic acid inhibited thapsigargin-induced store-operated calcium entry (SOCE) in a dose-dependent manner in Jurkat T cells (Carrillo et al., 2017).

In neutrophils, several studies have shown that oleic acid has activated different intracellular signaling pathways, such as intracellular calcium, PKC, PLC, ERK1/2, and p38 MAPK phosphorylation, Akt phosphorylation, and NF-κB activation (Carrillo et al., 2011; Hidalgo et al., 2011; Manosalva et al., 2015; Mena et al., 2016). In addition, the participation of PLC and PKC in intracellular calcium mobilization, and MMP-9 and ROS production was demonstrated. ERK1/2, p38 MAPK, and PI3K control NF-κB activation induced by oleic acid, and ERK1/2 participates in MMP-9 granules release (Manosalva et al., 2015; Mena et al., 2016).

Finally, a study showed that oleic acid could also affect NF-κB in macrophages, increasing its activation in macrophage J774 cells (de Lima et al., 2006).

### Linoleic Acid

Currently, there are no studies showing signaling pathways activated by linoleic acid in T-cells. In contrast, in neutrophils, some studies have described the signaling induced by linoleic acid. Manosalva et al. (2015) showed that linoleic acid induces intracellular calcium mobilization through PLC activation. Linoleic acid also induced the phosphorylation of ERK1/2, p38 MAPK and Akt, and reduced the levels of IκBα, which was dependent on the proteins described above (Mena et al., 2016). PLC, PKC, and ERK1/2 controlled MMP-9 release, PLC and PKC controlled ROS production, and NF-κB participated in COX-2 expression in bovine neutrophils (Manosalva et al., 2015; Mena et al., 2016). In macrophages, most studies have assessed the effect of linoleic acid-derived metabolites or conjugated linoleic acids, and only few studies have observed the effects of linoleic acid. These studies demonstrated that linoleic acid reduced the phosphorylation of MAPK (ERK1/2, JNK, and p38 MAPK) induced by stimuli, such as LDL or LPS, and the NF-κB activation in macrophages (Rahman et al., 2006; Stachowska et al., 2011; Kim et al., 2020).

### Palmitic Acid

As mentioned above, palmitic acid exerts its pro-inflammatory effect in T cells through the expression of SLAMF3. In addition, Zhou et al. (2019) demonstrated that palmitic acid activates the STAT5 and PI3K/Akt pathways, which control the expression of SLAMF3, thus proposing a mechanism by which fatty acids can promote inflammation in obesity or type 2 diabetes. Another study demonstrated that palmitic acid also activates the classical MAPK (JNK, ERK1/2, and p38) and the Akt pathway in Jurkat T cells (Takahashi et al., 2012).

The effect of palmitic acid on signaling pathway activation in neutrophils has been poorly studied. Khan et al. (2018) observed that palmitic acid induced NETosis dependent on NADPH oxidase, which required the activation of ERK and Akt.

The signaling pathways activated by palmitic acid in macrophages have been mainly linked to some TLR receptors and their signaling pathways, because studies on different cells have demonstrated the participation of these receptors in the effects induced by palmitic acid (reviewed in Korbecki and Bajdak-Rusinek, 2019). Studies on macrophages have shown that palmitic acid induces the activation and dimerization of TLR2/TLR1, TLR2/TLR6, and TLR4 (Huang et al., 2012; Hu et al., 2020), and induces the formation of the complex involving MD2/TLR4/MyD88, which is essential to induce cytokine production in mouse primary macrophages (Wang et al., 2017). Contrary to these lines of evidence, Lancaster et al. (2018) argued against these observations. They demonstrated through multiple experimental approaches that TLR4 is not a receptor for palmitic acid but that it indirectly regulates palmitic acid-induced inflammation as TLR4-dependent priming is a prerequisite for palmitic acid to induce inflammation (Lancaster et al., 2018). The authors observed that palmitic acid induce the phosphorylation of JNK and MKK4/7 after 4 h of treatment, but the pharmacological inhibition of TLR4 did not reduce JNK phosphorylation. Palmitic acid does not induce TLR4 dimerization or endocytosis (Lancaster et al., 2018).

### Omega-3: Docosahexaenoic Acid and Eicosapentaenoic Acid

The elucidation of signaling pathways modulated by omega-3 fatty acids has contributed to understanding the mechanisms of T-cell activation and the immunosuppressive effect of these fatty acids. Denys et al. (2005) demonstrated the inhibitory action of DHA on PMA-induced plasma membrane translocation of PKC-alpha and-epsilon and nuclear translocation of NF-κB in Jurkat T-cells. Furthermore, DHA exerts inhibitory effects on T-cell proliferation and increases free intracellular calcium through TRP3 and TRP6 calcium channels; however, it also reduces the expression of TRP3 and TRP6 (Saidi et al., 2020). These results suggest that the anti-proliferative effect of DHA might be dependent on the modulation of TRP3 and TRP6 channels.

In neutrophils, DHA also induced intracellular calcium mobilization in the absence of a stimulus, but experiments in the presence of an inflammatory stimulus showed that DHA reduced calcium influx and NF-κB activation (Fickl et al., 2005; Olmo et al., 2019). Several defensive functions of neutrophils are calcium-dependent; therefore, these findings suggest that DHA might modulate calcium-dependent functions to orchestrate an adequate innate immune response. However, more studies are required.

DHA and EPA also inhibit LPS-activated NF-κB in macrophages. DHA stimulates the expression, phosphorylation, and activity of α1AMPK, and induces the overexpression of SIRT1 and the deacetylation of p65 NF-κB, thereby regulating macrophage inflammation (Xue et al., 2012). In addition, DHA and EPA reduced the phosphorylation of the MAPK p38, ERK and JNK, p65 NF-κB, and the IκB degradation induced by RANKL in RAW264.7 macrophages, with a more potent effect observed with DHA compared with EPA (Rahman et al., 2008; Kasonga et al., 2019). DHA attenuated the JNK and Akt pathways activated by LPS in RAW264.7 macrophages (Li et al., 2013). Oh et al. (2010) unveiled a mechanism by which DHA exerts its anti-inflammatory effects. They demonstrated that DHA reduced the phosphorylation of TAK1, MKK4, and JNK, and decreased NF-κB activation and the release of TNF-α, IL-6, and MCP-1 in RAW264.7 and primary macrophages. Thus, the authors described a new anti-inflammatory mechanism of DHA.

**Figure 1:**
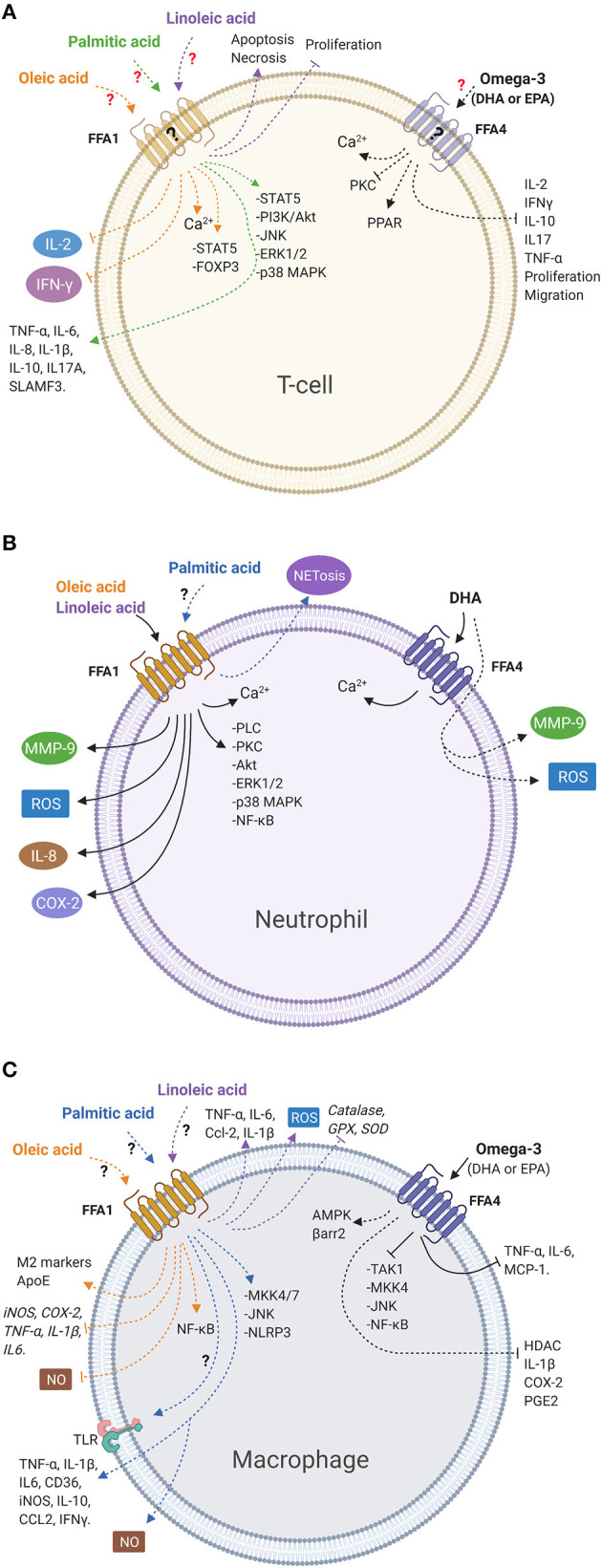
LCFAs activate signaling pathways and mediate immune cell functions. **(A)** Oleic and palmitic acids activate intracellular signaling pathways. Oleic acid reduces the release of cytokines, whereas palmitic acid increases the release of cytokines. Linoleic acid reduces viability and inhibits proliferation in T-cells. However, there is no evidence of the participation of the FFA1 receptor in those responses in this cell type. Omega-3 fatty acids inhibit cytokines release, migration, and proliferation. However, the participation of the FFA4 receptor has not been demonstrated in these cells. **(B)** Oleic and linoleic acids stimulate different signaling pathways and release inflammatory products *via* the FFA1 receptor in bovine neutrophils. Palmitic acid increases NETosis. However, the role of the FFA1 receptor has not been demonstrated. DHA induces intracellular calcium *via* the FFA4 receptor in neutrophils and increases release of inflammatory products, independent of the FFA4 receptor. **(C)** Oleic, linoleic, and palmitic acids activate signaling pathways and stimulate or inhibit the release of inflammatory mediators in macrophages. However, the participation of the FFA1 receptor has not been described yet. Omega-3 fatty acids inhibit intracellular signaling and release of inflammatory products through the FFA4 receptor in macrophages. Solid lines indicate cellular responses induced by fatty acids in which the participation of the FFA receptor has been demonstrated. Dashed lines represent responses induced by fatty acids where there is no evidence of the participation of the FFA receptors. Question marks indicate that FFA receptor involvement has not been demonstrated or FFA receptors have not been detected (created with BioRender.com).

A summary of the signaling pathways and effects induced by LCFAs, as well as the participation of the FFA1 and FFA4 receptors, is presented in Figure 1.

## Long-Chain Fatty Acid Receptors in Immune Cells

Progress has been made in elucidating the role of LCFAs in cellular functions based on the discovery of LCFA receptors. FFA1 (GPR40) and FFA4 (GPR120) are G protein-coupled receptors (GPCRs) for LCFAs, while other FFA receptors (FFA3/GPR41 and FFA2/GPR43 receptors) bind to short-chain fatty acids. The FFA1 and FFA4 receptors have been mostly studied in pancreatic cells, intestinal cells, and heterologous expression systems, owing to their relevance in diabetes and obesity. However, some studies have been performed on immune cells, and they have expanded the knowledge on the roles of LCFAs and inflammatory mechanisms. The following subsections will address the pharmacology of the FFA1 and FFA4 receptors and then the role of these receptors in immune cells.

### Pharmacology of the FFA1 and FFA4 Receptors

The FFA1 receptor was the first deorphanized GPCR for fatty acids (Sawzdargo et al., 1997). It is highly expressed in human β-pancreatic cells (Itoh et al., 2003) and gut enteroendocrine responsible for glucagon-like peptide-1 (GLP-1) and peptide hormone YY (PYY) (Milligan et al., 2017). In addition, the FFA1 receptor is expressed in several tissues, such as those of the heart, skeletal muscle, liver, bone, brain, epithelial mammary cells, endothelial cells, macrophages, monocytes, and neutrophils (Briscoe et al., 2003; Kotarsky et al., 2003; Yonezawa et al., 2004, 2008; Cornish et al., 2008; Manosalva et al., 2015; Loaiza et al., 2016; Agrawal et al., 2017; Souza et al., 2020). Since FFA1 receptor agonists in β-pancreatic cells increase glucose-dependent insulin release (Briscoe et al., 2003; Itoh et al., 2003), basic FFA1 receptor-related research has mainly focused on investigating its potential use in diabetes mellitus therapy (Burant, 2013; Mohammad, 2016).

#### Agonists of the FFA1 Receptor

Both medium-chain fatty acids (MCFA) and LCFAs are natural ligands of the FFA1 receptor, such as oleic, linoleic, and palmitic acids (Table 1) (Briscoe et al., 2003). In addition, diverse synthetic agonists have been developed mainly for therapeutic purposes. However, they have also been useful for the pharmacological characterization of the FFA1 receptor. Since pharmacological studies on the FFA1 receptor and its natural ligands have been carried out in heterologous expression systems, and the effects of oleic, linoleic, and palmitic acids on immune cells have been reviewed above, we will review some synthetic agonists that have been described for the FFA1 receptor (Table 1).

**Table 1:** Ligands for the FFA1 receptor, signaling pathways, and effects on immune cells.

**Action**	**Ligand**	**Effects on immune cells**	**References**
Natural agonist	Oleic acid	↑ FOXP3 and p-STAT5; ↓ SOCE, IL-2, IFN-γ, proliferation in T-cells.	Verlengia et al., 2003; Carrillo et al., 2017; Pompura et al., 2020
		↓ migration; ↑ calcium, PKC, PLC, ERK1/2, p38 MAPK, Akt, NF-κB, ROS, MMP-9, VEGF, IL-1β, IL-8, COX-2, NETs, and ATP in neutrophils.	Hatanaka et al., 2006; Pereira et al., 2008; Carrillo et al., 2011; Hidalgo et al., 2011; Reyes-Quiroz et al., 2014; Manosalva et al., 2015; Mena et al., 2016; Medeiros-de-Moraes et al., 2018; Alarcon et al., 2020
		↑ M2 markers, NF-κB, apoE; ↓ iNOS, COX-2, TNF-α, IL1β, IL6, and apoptosis in macrophages.	Huang et al., 2004; de Lima et al., 2006; Camell and Smith, 2013; Kim et al., 2017; Karasawa et al., 2018; Muller et al., 2019
	Linoleic acid	↓ proliferation in T-cell; ↑ apoptosis in T-cell.	Jeffery et al., 1996, 1997; Cury-Boaventura et al., 2004
		↑ calcium, PLC, ERK1/2, p38 MAPK, Akt, NF-κB, ROS, TX, TNF-α, MMP-9, COX-2, IL-8, NETs, IL-1β, CINC-2αβ, VEGFα in neutrophils	Vaughan and Walsh, 2005; Pereira et al., 2008; Rodrigues et al., 2010; Hidalgo et al., 2011; Manosalva et al., 2015; Mena et al., 2016; Alarcon et al., 2020
		↓ IL-1β, IL-6, IL-10, ERK1/2, JNK, p38 MAPK, NF-κB; ↑ TNF-α, IL-6, Ccl2 and IL-1β and ROS in macrophages	Rahman et al., 2006; Rybicka et al., 2011; Stachowska et al., 2011; Magdalon et al., 2012; Kain and Halade, 2019; Kim et al., 2020
	Palmitic acid	↑ STAT5, PI3K/Akt, JNK, ERK1/2, SLAMF3, IFN-γ, TNF-α, IL-6, IL-8, IL-1β, IL-2, IL-10, proliferation, SLAMF3, IL17A in T-cells.	Karsten et al., 1994; Lima et al., 2002; Tinahones et al., 2004; Stentz and Kitabchi, 2006; Gorjao et al., 2007; Takahashi et al., 2012; Zhou et al., 2019; Torres-Hernandez et al., 2020
		↑ ERK1/2, Akt, NETs, ROS, chemotaxis in neutrophils.	Wanten et al., 2002; Khan et al., 2018; Tam et al., 2020
		↑ adhesion, apoptosis, NLRP3, NO, IL-1β, IL-6, TNF-α, NF-κB, JNK, MKK4/7, IL-10, CCL2, IFN-γ M1 polarization, CD36; ↓ phagocytosis in macrophages.	Calder et al., 1990a; de Lima et al., 2006; Kim et al., 2017; Lu et al., 2017; Wang et al., 2017, 2021; Karasawa et al., 2018; Lancaster et al., 2018; Korbecki and Bajdak-Rusinek, 2019; Fu et al., 2020; Sano et al., 2020; Youk et al., 2021
Synthetic agonist	GW9508	↑ ROS, MMP-9, COX-2, IL-8, chemotaxis in neutrophils.	Hidalgo et al., 2011; Manosalva et al., 2015; Mena et al., 2016; Souza et al., 2020
		↑ PLC, PLA2, AMPK, AA; ↓ IL-6 in macrophages.	Liu et al., 2014; An et al., 2020
Antagonist	GW1100	↓ MMP-9, ROS, IL-8, COX-2 in neutrophils.	Manosalva et al., 2015; Mena et al., 2016

*↑, increases; ↓, decreases*.

GW9508 {4-[[(3-Phenoxyphenyl)methyl]amino]benzene propanoic acid} is a partial agonist of the FFA1 receptor (pEC50 = 7.32), being 100-fold more selective for the FFA1 receptor than for the FFA4 receptor (pEC50 = 5.46) (Briscoe et al., 2006). However, it has also been used as an FFA4 receptor agonist in FFA4 receptor-expressing cells (Son et al., 2021). In bovine neutrophils, GW9508 and natural ligands increase the release of ROS and MMP-9, and the expression of COX-2 and IL-8, suggesting a potential role of the FFA1 receptor in innate immune response and inflammation (Hidalgo et al., 2011; Manosalva et al., 2015; Mena et al., 2016). In human neutrophils, the FFA1 receptor is upregulated upon activation with pro-inflammatory stimuli, such as PAF and LTB4, and GW9508 enhances IL-8-induced chemotaxis and prolongs human neutrophil lifespan (Souza et al., 2020). In macrophages, GW9508 (used at 100 or 200 μM) induced the expression of ABC transporters and activated intracellular signaling pathways, such as PLC and AMPK, PLA2, and arachidonic acid release, and reduced LPS-induced IL-6 secretion (Liu et al., 2014; An et al., 2020).

Partial agonists, TAK-875 (Negoro et al., 2010) and AMG 837 (Hauge et al., 2015), and full agonists, AM-1638 (Brown et al., 2012; Lin et al., 2012) and AM-5262 (Wang et al., 2013), have been described. Radioligand interaction binding experiments suggest that full agonists do not interact with the binding site of partial agonists. However, positive cooperativity has been observed in *in vitro* and *in vivo* functional assays (Lin et al., 2012; Xiong et al., 2013). TAK-875 (Fasiglifam) was one of the first synthetic agonists used to prove the utility of the FFA1 receptor in the treatment of type 2 diabetes (Kaku et al., 2015). TAK-875 activates the recruitment of β-arrestin-2 with higher efficacy and potency (EC50: 54.7 nM) compared with oleic and palmitic acids (EC50: 58.4 and 42.4 μM, respectively) (Mancini et al., 2015). In contrast, oleic and palmitic acids show higher potency and efficacy in activating the Gαq/11 signaling pathway compared with TAK-875, acting as a partial agonist and revealing the complexity of FFA1 receptor signal transduction (Mancini et al., 2015). TAK-875, as an allosteric modulator, potentiates fatty acid-induced insulin secretion, and fatty acids increase the activity of TAK-875 (Yabuki et al., 2013). Even though TAK-875 shows clinical efficacy in patients with type 2 diabetes, side effects, such as nasopharyngitis, upper respiratory tract inflammation, and liver toxicity, have been described (Kaku et al., 2015; Suckow and Briscoe, 2017). AMG837 (Amgen) is a β-substituted phenylpropanoic acid and potent FFA1 receptor agonist that has been proposed as a candidate for type 2 diabetes therapy (Houze et al., 2012; Luo et al., 2012). However, phase 1 clinical trials have been discontinued probably because of concerns over toxicity (Suckow and Briscoe, 2017). AM-1638 is a back-up candidate of AMG837 reported as an FFA1 receptor full agonist (Lin et al., 2012). In COS7 cells transiently transfected with human the FFA1 receptor, AM-1638 activates Gαq and Gαs, while alpha-linolenic acid and DHA only activate Gαq, suggesting complex intracellular signaling induced by AM-1638 that would contribute to the higher efficacy observed in incretin release (Hauge et al., 2015). Another similar derivative, AM-5262, showed improved efficacy but similar binding properties and signaling pathways compared with AM-1638 (Wang et al., 2013; Hauge et al., 2015).

LY2881835 (Eli Lilly), a spiro [indene-1, 4-peperdine], shows a partial agonist effect on the FFA1 receptor with an EC50 of 164 nmol/L in calcium flux assay but shows a full agonist effect in β-arrestin recruitment assays (EC50: 8.7 nmol/L) (Chen et al., 2016).

Clinical trials using agonist molecules for the FFA1 receptor had to be abandoned or stopped because of adverse effects, such as liver injury (Menon et al., 2018).

#### Antagonists of the FFA1 Receptor

Unlike the evolution and development of FFA1 receptor agonists, only few molecules have been studied as antagonists (Table 1). GW1100 was the first FFA1 receptor antagonist that reduced the calcium increase induced by GW9508 and linoleic acid in HEK-293 cells expressing the human FFA1 receptor (pIC50 values of 5.99 ± 0.03 and 5.99 ± 0.06, respectively). However, it did not show this response in cells expressing the FFA4 receptor (Briscoe et al., 2006). GW1100 reduced the FFA1 receptor ligand-induced release of MMP-9 granules, ROS production, IL-8 release, and COX2 expression in neutrophils (Manosalva et al., 2015; Mena et al., 2016).

DC260126 containing a sulfonamide structure inhibited intracellular calcium responses induced by several FFA receptor ligands and reduced ERK1/2 phosphorylation stimulated by linoleic acid in FFA1 receptor-expressing CHO cells (Hu et al., 2009). In addition, long-term DC1260126 treatment inhibited glucose-stimulated insulin secretion and serum insulin levels and protected against pancreatic β-cell dysfunction in obese diabetic db/db mice (Sun et al., 2013).

The pyrimidinyl hydrazone ANT-203 was developed by the high-throughput screening of HEK293 cells expressing the human FFA1 receptor. ANT-203 showed an anti-apoptotic effect on MIN6 cells (IC50 = 251 nM). However, it demonstrated low solubility and bioavailability (Kristinsson et al., 2013).

The FFA4 receptor is the second deorphanized receptor for LCFAs, which is abundantly expressed in the intestine and other tissues, such as those of the lungs, thymus, brain, adrenal gland, spleen, and placenta (Hirasawa et al., 2005; Lager et al., 2014). It is also expressed in the adipose tissue, pro-inflammatory macrophages, and neutrophils (Oh et al., 2010; Olmo et al., 2019). LCFAs are natural ligands for the FFA4 receptor, and omega-3 fatty acids demonstrate higher potency compared with other fatty acids (Hirasawa et al., 2005). The regulation of the release of incretin glucagon-like peptide-1 (GLP) secretion was the initial function observed for the FFA4 receptor after stimulation with PUFA (Hirasawa et al., 2005). Subsequently, it was described a function of insulin-sensitization and reduction of the inflammation induced by macrophages, which could explain their anti-diabetic effects (Oh et al., 2010).

#### Agonists of the FFA4 Receptor

Several synthetic agonists of the FFA4 receptor have been studied in *in vitro* and *in vivo* assays (Table 2). NCG21 [4-{4-[2-(phenyl-pyridin-2-yl-amino)-ethoxy]-phenyl}-butyric acid], is a selective agonist of the FFA4 receptor obtained from peroxisome proliferator-activated receptor-γ (PPAR-γ) agonist derivatives (Suzuki et al., 2008). NCG21 has 10-fold higher selectivity for the FFA4 receptor over the FFA1 receptor in an intracellular calcium assay performed in HEK293 cells expressing the FFA4 receptor (Suzuki et al., 2008).

**Table 2:** Ligands for the FFA4 receptor, signaling pathways, and effects on immune cells.

**Action**	**Ligand**	**Effects on immune cells**	**References**
Natural agonist	DHA, EPA	↓ proliferation, migration, IL-2, IFN-γ, IL-17, IL-10, NF-κB, PKC; ↑ calcium, PPAR, apoptosis, ROS in T-cells.	Verlengia et al., 2004; Denys et al., 2005; Yessoufou et al., 2009; Unoda et al., 2013; Zhelev et al., 2016; Cucchi et al., 2020; Saidi et al., 2020
		↑ phagocytosis, TNF-α, IL-1β, MMP-9, ROS, calcium; ↓ IL-8, ROS, calcium and NF-κB in neutrophils.	Fickl et al., 2005; Gorjao et al., 2006; Pisani et al., 2009; Paschoal et al., 2013; Olmo et al., 2019
		↓ HDACs, Inflammasome, IL-1, TAK1, MKK4, NF-κB, p38 MAPK, ERK, JNK;↑ M2 macrophages.	Rahman et al., 2008; Oh et al., 2010; Xue et al., 2012; Li et al., 2013; Pham et al., 2018; Kasonga et al., 2019; Kawano et al., 2019; Wierenga et al., 2019
Synthetic agonist	TUG-891	↑ calcium, MMP-9 and ROS in neutrophils.	Olmo et al., 2019
		↑ M2 markers; ↓ M1 markers, motility and phagocytosis in macrophages.	Hudson et al., 2013; Wang et al., 2019a; Zhao et al., 2019; Su et al., 2020
	Grifolic acid	↓ viability, ATP and MMP in macrophages.	Zhao et al., 2018
	GSK 137647	↓ MMP-9, MMP-3, TIMP-1 and migration in macrophages.	Hasan et al., 2017
	Compound A	↓ NF-κB, TNF-α, IL-6 and chemotaxis, in macrophages.	Oh et al., 2014
	KDT501	↓ IL-6, IL-10, MCP-1 and RANTES in macrophages.	Konda et al., 2014
Antagonist	AH7614	↓ Calcium and ROS in neutrophils.	Olmo et al., 2019

*↑, increases; ↓, decreases*.

Grifolic acid {2,4-dihydroxy-6-methyl-3-[(2E,6E)-3,7,11-trimethyl-2,6,10-dodecatrien-1-yl]-benzoic acid}, is a partial agonist of the FFA4 receptor that activates ERK and augments intracellular calcium concentrations in cells expressing the FFA4 receptor but not the FFA1 receptor (Hara et al., 2009). Grifolic acid reduced cell viability, ATP levels, and mitochondrial membrane potential in macrophages (Zhao et al., 2018).

TUG-891, 3-(4-{[5-fluoro-2-(4-methylphenyl)phenyl]methoxy}phenyl)propanoic acid, is a potent and selective human FFA4 receptor agonist showing calcium mobilization, β-arrestin1 and β-arrestin 2 recruitment, and ERK phosphorylation properties, similar to those observed with alpha-linolenic acid (αLA) in human FFA4 receptor-expressing cells (Hudson et al., 2013). However, its selectivity for the mouse FFA4 receptor has not been confirmed. Despite this, TUG-891 has been used as a tool for the study of the FFA4 receptor using siRNA technology in KO mice. TUG-891 induced intracellular calcium mobilization (EC50 73 μM), increased MMP-9 release, and ROS production in bovine neutrophils (Olmo et al., 2019), and upregulated the expression of M2 marker macrophages CD206 and arginase-1, while reducing the expression of M1 markers IL-6 and TNF-α (Wang et al., 2019a). TUG-891 inhibited the release of TNFα induced by LPS in RAW264.7 macrophages (Hudson et al., 2013). TUG-891 administration in drinking water ameliorated inflammation in visceral white adipose tissue and insulin resistance (Gozal et al., 2016). TUG-891 interferes with the motility and phagocytosis of alveolar macrophages *via* a Gq protein-PLC-calcium release pathway (Su et al., 2020). LPS treatment counteracted the effects of TUG-891 on the inhibition of phagocytosis by alveolar macrophages interfering with the expression of the FFA4 receptor (Zhao et al., 2019).

GSK137647A [4-methoxy-N-(2,4,6-trimethylphenyl) benzenesulfonamide], is the first non-carboxylic FFA4 receptor agonist (pEC50 = 6.3) demonstrating 50-fold higher selectivity for the FFA4 receptor over the FFA1 receptor, with similar responses across species (Sparks et al., 2014). This FFA4 receptor agonist increases the mineralization of differentiated osteoblasts and reduces the adipogenic differentiation of bone mesenchymal stem cells (Wang et al., 2020). GSK137647A restored pancreatic duodenal homeobox-1 (PDX1) expression levels and β-cell function by inhibiting lipotoxicity induced by palmitic acid, interfering with an increase in the expression of pro-inflammatory chemokines and activation of NF-κB, JNK1/2, and p38 MAPK signaling pathways (Wang et al., 2019b). GSK137647 significantly reduced the mRNA levels and secretion of IL-6 and CCL2 in 3T3-L1 adipocytes, both basal and those stimulated by LPS (Hasan et al., 2017). GSK137647 reduced the production of extracellular matrix-modulating factors induced by adipocytes in macrophages and reduced the migration of macrophages (Hasan et al., 2017).

Compound A was developed as an orally available, selective FFA4 receptor agonist (EC50 = ~0.35 μM) and shows anti-inflammatory effects in macrophages *in vitro*. In HFD-fed obese mice, the compound improved glucose tolerance and insulin sensitivity and decreased hyperinsulinemia and hepatic steatosis (Oh et al., 2014). Compound A reduced 2,4-dinitrochlorobenzene-induced atopic dermatitis that is mediated through FFA4 receptor activation and Foxp3+ Treg increase in mice (Son et al., 2020). However, other studies have suggested that therapies based only on FFA4 receptor activation would not be helpful in the treatment of inflammatory diseases. They observed that Compound A did not modify the course of Aldara-induced psoriasis-like dermatitis, K/BxN serum transfer arthritis, or antibody transfer pemphigoid disease-like dermatitis (Wannick et al., 2018). Metabolex 36 and metabolex compound B have been used as FFA4 receptor agonists. Metabolex 36 shows an EC50 value of 1.17 μM with the FFA4 receptor and >100 μM with the FFA1 receptor (Stone et al., 2014), whereas compound B showed EC50 = 15 nM on the FFA4 receptor and 1,000-fold selectivity over the FFA1 receptor in inositol triphosphate accumulation assays performed with FFA4 receptor-transfected COS7 cells (Engelstoft et al., 2013). Compound B inhibited basal ghrelin secretion from primary gastric mucosal cells and somatostatin release from primary gastric epithelial cells; however, these effects were not observed in cells derived from FFA4 receptor KO mice (Engelstoft et al., 2013; Egerod et al., 2015). TUG-1197 was developed from a series of cyclic sulfonamide FFA4 receptor agonists, with pEC50 = 6.6 and 6.8 as demonstrated in calcium mobilization assays performed for examining human and murine FFA4 receptors, respectively (Azevedo et al., 2016). KDT501 is an isohumulone structure derived from hop extracts and aids in the control of impaired glucose and insulin regulation in patients with insulin resistance. The agonist activity on the FFA4 receptor was estimated at EC50 = 30.3 μM with weak and partial agonism on PPARγ. KDT501 improved insulin sensitivity and glucose regulation and reduced the expression of LPS-stimulated inflammatory mediators MCP-1, RANTES, and IL-6 in monocytic THP-1 cells (Konda et al., 2014).

#### Antagonists of the FFA4 Receptor

AH7614 was first described as an antagonist of the FFA4 receptor since it inhibited FFA4 receptor activation by GSK137647A and linoleic acid; however, it did not demonstrate appropriate properties for utilization in an *in vivo* setting (Sparks et al., 2014). AH7614 was proposed as a negative allosteric modulator of the FFA4 receptor that inhibits signaling induced by a range of FFA4 receptor agonists, such as TUG-891, TUG-1197, GSK137647A, and compound A (Watterson et al., 2017). AH7614 reduced the intracellular calcium mobilization and ROS production induced by TUG-891 in bovine neutrophils (Table 2) (Olmo et al., 2019). Arachidonic acid increased intracellular calcium in cells expressing green fluorescent protein-tagged human FFA4 receptors and was inhibited by AH7614 (Villegas-Comonfort et al., 2017). The protective effect of DHA on hepatic steatosis was diminished by treatment with AH7614 and not observed in primary hepatocytes derived from FFA4 receptor-deficient mice (Kang et al., 2018).

### Role of the FFA1 and FFA4 Receptors on Immune Cells

Since most studies examining the pharmacology and expression of the FFA1 and FFA4 receptors have been performed in pancreatic cell, intestinal cell, and heterologous expression systems, they have also described some intracellular signaling pathways and cellular effects mediated by the FFA receptors in those cells. On the contrary, only few studies have demonstrated the presence and role of the FFA1 or FFA4 receptors in neutrophils or macrophages.

#### The FFA1 Receptor

The presence of the FFA1 receptor has been confirmed in neutrophils, but opposite results about its expression in macrophages have been observed (Cornish et al., 2008; Oh et al., 2010; Manosalva et al., 2015; Souza et al., 2020). A study showed expression of the FFA1 receptor, although at low levels, but another study did not detect the FFA1 receptor in RAW264.7 macrophages (Cornish et al., 2008; Oh et al., 2010). In contrast, the FFA1 receptor has not been detected in T cells (Briscoe et al., 2003).

A reduced number of studies have evaluated the participation of the FFA1 receptor in neutrophil functions and intracellular signaling induced by LCFA. The FFA1 receptor is activated by oleic and linoleic acids, and the synthetic agonist GW9508 in neutrophils, as demonstrated by intracellular calcium assay (Carrillo et al., 2011; Hidalgo et al., 2011; Manosalva et al., 2015). The increase in intracellular calcium induced by linoleic acid and GW9508 is essentially mediated by the FFA1 receptor, as was demonstrated through assays in CHO cells transfected with the bovine FFA1 receptor. Also, assays performed in bovine neutrophils treated with the FFA1 receptor antagonist GW1100 showed whole inhibition of the intracellular calcium mobilization induced by linoleic acid and GW9508. However, when oleic acid was assessed in neutrophils treated with GW1100, only partial inhibition was observed, suggesting the participation of other mechanisms in intracellular calcium mobilization. The FFA1 receptor controls the activation of several intracellular signaling pathways (PKC, PLC, phosphorylation of ERK1/2, p38 MAPK, and Akt, and NF-κB activation) induced by oleic and linoleic acids (Carrillo et al., 2011; Hidalgo et al., 2011; Manosalva et al., 2015; Mena et al., 2016). However, results similar to those of calcium assays were observed when oleic acid was used in some experiments of intracellular signaling or neutrophil functions in the presence of GW1100, suggesting that oleic acid might exert its effects partially *via* the FFA1 receptor. In addition, the participation of the FFA1 receptor in IL-8 and COX-2 expression, MMP-9 release, and ROS production were demonstrated (Manosalva et al., 2015; Mena et al., 2016). Recently, Souza et al. (2020) suggested the participation of the FFA1 receptor in chemotaxis and phagocytosis, in *in vitro* and *in vivo* assays using the synthetic FFA1 receptor agonist GW9508. In summary, all these findings argue that the FFA1 receptor may play a host-protective role against infection or tissue injury. However, we did not rule out that LCFAs can produce their effect through additional mechanisms, such as incorporation into membrane phospholipids or diffusion through the membrane to exert their effects intracellularly, mechanisms which remain to be demonstrated. More studies on the participation of the FFA1 receptor in other neutrophil functions, potential mechanisms activated by LCFAs, and the *in vivo* role of the FFA1 receptor are necessary.

#### The FFA4 Receptor

The presence of the FFA4 receptor in T cell has not been studied yet. In contrast, evidence supports the presence of the FFA4 receptor in neutrophils and macrophages (Oh et al., 2010; Olmo et al., 2019). DHA and the synthetic agonist TUG-891 induced intracellular calcium mobilization in bovine neutrophils, which was partially inhibited by AH7614 (antagonist of the FFA4 receptor), suggesting the participation of this receptor in calcium mobilization (Olmo et al., 2019). In addition, as described above, DHA also stimulates MMP-9 granules release and ROS production in neutrophils However, these responses were not inhibited by AH7614, which strongly suggests the involvement of other mechanisms in the action of DHA, such as incorporation into the cellular membrane and diffusion through it.

In macrophages, Oh et al. (2010) unveiled a mechanism by which DHA exerts its anti-inflammatory effects. They demonstrated that the inhibitory effect of DHA on intracellular signaling pathways (TAK1, MKK4, JNK, NF-κB) and release of TNF-α, IL-6, and MCP-1 in macrophages are mediated by the FFA4 receptor and the activation of β-arrestin2. Thus, the authors described a new anti-inflammatory mechanism of DHA mediated by the FFA4 receptor.

Overall, the findings described above support the protective and anti-inflammatory role of omega-3 fatty acids mediated by the FFA4 receptor. However, it is important to consider that DHA and EPA also have a key pro-resolution role during the inflammatory process through the formation of specialized pro-resolving mediators (SPM). Therefore, the FFA4 receptor should not be considered as a single anti-inflammatory mechanism.

## Conclusions

The importance of LCFAs has been extensively demonstrated in metabolic diseases, but to a lesser extent in inflammation. While there is evidence of the effects of LCFAs on immune cells in higher or lower magnitude depending on the fatty acid, the role of the FFA1 and FFA4 receptors in these cells is yet to be elucidated. In T-cells, oleic and palmitic acids activate intracellular signaling pathways. Oleic acid reduces the release of cytokines, and palmitic acid increases cytokine release. Linoleic acid reduces viability and inhibits proliferation, whereas omega-3 fatty acids inhibit cytokine release, migration, and proliferation in T-cells. However, in T-cells, the FFA1 and FFA4 receptors have not been detected, which strongly suggests that LCFAs induce their effects through other mechanisms. In neutrophils, oleic and linoleic acids stimulate different signaling pathways and release inflammatory products through the FFA1 receptor. Palmitic acid increases NETosis; however, the role of the FFA1 receptor has not been demonstrated. DHA induces intracellular calcium *via* the FFA4 receptor in neutrophils and increases the release of inflammatory products, independent of the FFA4 receptor. In macrophages, oleic, linoleic, and palmitic acids activate signaling pathways and stimulate or inhibit the release of inflammatory mediators; however, the participation of the FFA1 receptor has not been described yet. Omega-3 fatty acids inhibit intracellular signaling and release inflammatory products through the FFA4 receptor.

In summary, the FFA1 receptor has been studied in neutrophils, but there is no evidence demonstrating its role in macrophages and T-cells. In contrast, the role of the FFA4 receptor in cellular activation has been demonstrated in macrophages and, to a lesser extent, in neutrophils, but not in T-cells. Although several effects of LCFAs have been demonstrated, studies on intracellular signaling pathways activated by these fatty acids and the role of the FFA receptors are still incipient. It is necessary to continue studying the mechanisms by which LCFAs exert their effects and regulate the immune function; and more *in vivo* studies are necessary, because some contradictory results observed in *in vitro* assays can limit its usefulness. Also, *in vivo* studies that assess the potential useful of agonists or antagonists of the FFA1 and FFA4 receptors in inflammatory diseases are necessary. Although several studies have assessed the potential use of synthetic agonists of the FFA1 receptor for the treatment of metabolic diseases, they have not been successful because of some toxic effects reported. In contrast, natural agonists of the FFA receptors have not reported toxic effects at concentrations that are achieved in plasma. Therefore, we propose that the dietary supplementation with LCFA (in an adequate ratio) might be a useful tool to improve inflammatory diseases, which also would contribute to control metabolic diseases, thus regulating homeostasis immune and metabolic.

## Author Contributions

MH, MC, and RB drafted the manuscript. MH wrote the manuscript (sections Introduction, Effects of LCFAs on Immune Cells, Intracellular Signaling Pathways Activated by Long-Chain Fatty Acids in Immune Cells, and Conclusions, and the tables, and figure). MC wrote section Role of the FFA1 and FFA4 Receptors on Immune Cells. RB wrote section Pharmacology of the FFA1 and FFA4 Receptors. All the authors critically discussed and approved the submitted manuscript.

## Conflict of Interest

The authors declare that the research was conducted in the absence of any commercial or financial relationships that could be construed as a potential conflict of interest.

## References

[B1] Abdelmagid S. A. Clarke S. E. Nielsen D. E. Badawi A. El-Sohemy A. Mutch D. M.. (2015). Comprehensive profiling of plasma fatty acid concentrations in young healthy Canadian adults. PLoS ONE 10:e0116195. 10.1371/journal.pone.0116195 25675440 PMC4326172

[B2] Agrawal A. Alharthi A. Vailati-Riboni M. Zhou Z. Loor J. J. (2017). Expression of fatty acid sensing G-protein coupled receptors in peripartal Holstein cows. J. Anim. Sci. Biotechnol. 8:20. 10.1186/s40104-017-0150-z 28261474 PMC5331663

[B3] Akamatsu H. Niwa Y. Matsunaga K. (2001). Effect of palmitic acid on neutrophil functions *in vitro*. Int. J. Dermatol. 40, 640–643. 10.1046/j.1365-4362.2001.01292.x 11737424

[B4] Alarcon P. Manosalva C. Quiroga J. Belmar I. Alvarez K. Diaz G.. (2020). Oleic and linoleic acids induce the release of neutrophil extracellular traps via pannexin 1-dependent ATP release and P2X1 receptor activation. Front. Vet. Sci. 7:260. 10.3389/fvets.2020.00260 32582772 PMC7291836

[B5] Ambrozova G. Fidlerova T. Verescakova H. Koudelka A. Rudolph T. K. Woodcock S. R.. (2016). Nitro-oleic acid inhibits vascular endothelial inflammatory responses and the endothelial-mesenchymal transition. Biochim. Biophys. Acta 1860(11 Pt A), 2428–2437. 10.1016/j.bbagen.2016.07.010 27431604 PMC5010974

[B6] An T. Zhang X. Li H. Dou L. Huang X. Man Y.. (2020). GPR120 facilitates cholesterol efflux in macrophages through activation of AMPK signaling pathway. FEBS J. 287, 5080–5095. 10.1111/febs.15310 32243091

[B7] Arrington J. L. Chapkin R. S. Switzer K. C. Morris J. S. McMurray D. N. (2001a). Dietary n-3 polyunsaturated fatty acids modulate purified murine T-cell subset activation. Clin. Exp. Immunol. 125, 499–507. 10.1046/j.1365-2249.2001.01627.x 11531960 PMC1906146

[B8] Arrington J. L. McMurray D. N. Switzer K. C. Fan Y. Y. Chapkin R. S. (2001b). Docosahexaenoic acid suppresses function of the CD28 costimulatory membrane receptor in primary murine and Jurkat T cells. J. Nutr. 131, 1147–1153. 10.1093/jn/131.4.1147 11285317

[B9] Azevedo C. M. Watterson K. R. Wargent E. T. Hansen S. V. Hudson B. D. Kepczynska M. A.. (2016). Non-acidic free fatty acid receptor 4 agonists with antidiabetic activity. J. Med. Chem. 59, 8868–8878. 10.1021/acs.jmedchem.6b00685 27570890

[B10] Azrad M. Turgeon C. Demark-Wahnefried W. (2013). Current evidence linking polyunsaturated Fatty acids with cancer risk and progression. Front. Oncol. 3:224. 10.3389/fonc.2013.00224 24027672 PMC3761560

[B11] Bagga D. Wang L. Farias-Eisner R. Glaspy J. A. Reddy S. T. (2003). Differential effects of prostaglandin derived from omega-6 and omega-3 polyunsaturated fatty acids on COX-2 expression and IL-6 secretion. Proc. Natl. Acad. Sci. U.S.A. 100, 1751–1756. 10.1073/pnas.0334211100 12578976 PMC149905

[B12] Boden G. (2008). Obesity and free fatty acids. Endocrinol. Metab. Clin. North Am. 37, 635–646, viii–ix. 10.1016/j.ecl.2008.06.007 18775356 PMC2596919

[B13] Briscoe C. P. Peat A. J. McKeown S. C. Corbett D. F. Goetz A. S. Littleton T. R.. (2006). Pharmacological regulation of insulin secretion in MIN6 cells through the fatty acid receptor GPR40: identification of agonist and antagonist small molecules. Br. J. Pharmacol. 148, 619–628. 10.1038/sj.bjp.0706770 16702987 PMC1751878

[B14] Briscoe C. P. Tadayyon M. Andrews J. L. Benson W. G. Chambers J. K. Eilert M. M.. (2003). The orphan G protein-coupled receptor GPR40 is activated by medium and long chain fatty acids. J. Biol. Chem. 278, 11303–11311. 10.1074/jbc.M211495200 12496284

[B15] Brown S. P. Dransfield P. J. Vimolratana M. Jiao X. Zhu L. Pattaropong V.. (2012). Discovery of AM-1638: a potent and orally bioavailable GPR40/FFA1 full agonist. ACS Med. Chem. Lett. 3, 726–730. 10.1021/ml300133f 24900539 PMC4025659

[B16] Burant C. F. (2013). Activation of GPR40 as a therapeutic target for the treatment of type 2 diabetes. Diabetes Care 36(Suppl. 2), S175–179. 10.2337/dcS13-2037 23882043 PMC3920793

[B17] Burdge G. C. Lillycrop K. A. (2014). Fatty acids and epigenetics. Curr. Opin. Clin. Nutr. Metab. Care 17, 156–161. 10.1097/MCO.0000000000000023 24322369

[B18] Butler M. Huzel N. Barnabe N. (1997). Unsaturated fatty acids enhance cell yields and perturb the energy metabolism of an antibody-secreting hybridoma. Biochem. J. 322(Pt 2), 615–623. 10.1042/bj3220615 9065785 PMC1218234

[B19] Buttke T. M. (1984). Inhibition of lymphocyte proliferation by free fatty acids. I. Differential effects on mouse B and T lymphocytes. Immunology 53, 235–242. 6333382 PMC1454828

[B20] Calder P. C. Bond J. A. Harvey D. J. Gordon S. Newsholme E. A. (1990a). Uptake and incorporation of saturated and unsaturated fatty acids into macrophage lipids and their effect upon macrophage adhesion and phagocytosis. Biochem. J. 269, 807–814. 10.1042/bj2690807 2117922 PMC1131659

[B21] Calder P. C. Bond J. A. Newsholme E. A. (1990b). Fatty acid inhibition of lipopolysaccharide-stimulated B lymphocyte proliferation. Biochem. Soc. Trans. 18, 904–905. 10.1042/bst0180904 2083721

[B22] Camell C. Smith C. W. (2013). Dietary oleic acid increases m2 macrophages in the mesenteric adipose tissue. PLoS ONE 8:e75147. 10.1371/journal.pone.0075147 24098682 PMC3787090

[B23] Campoio T. R. Oliveira F. A. Otton R. (2011). Oxidative stress in human lymphocytes treated with fatty acid mixture: role of carotenoid astaxanthin. Toxicol. Vitro 25, 1448–1456. 10.1016/j.tiv.2011.04.018 21549829

[B24] Capo X. Martorell M. Sureda A. Tur J. A. Pons A. (2015). Effects of docosahexaenoic supplementation and *in vitro* vitamin C on the oxidative and inflammatory neutrophil response to activation. Oxid. Med. Cell. Longev. 2015:187849. 10.1155/2015/187849 25960826 PMC4417594

[B25] Carlsson J. A. Wold A. E. Sandberg A. S. Ostman S. M. (2015). The polyunsaturated fatty acids arachidonic acid and docosahexaenoic acid induce mouse dendritic cells maturation but reduce T-cell responses *in vitro*. PLoS ONE 10:e0143741. 10.1371/journal.pone.0143741 26619195 PMC4664484

[B26] Carrillo C. Del Mar Cavia M. Roelofs H. Wanten G. Alonso-Torre S. R. (2011). Activation of human neutrophils by oleic acid involves the production of reactive oxygen species and a rise in cytosolic calcium concentration: a comparison with N-6 polyunsaturated fatty acids. Cell. Physiol. Biochem. 28, 329–338. 10.1159/000331749 21865741

[B27] Carrillo C. Giraldo M. Cavia M. M. Alonso-Torre S. R. (2017). Effect of oleic acid on store-operated calcium entry in immune-competent cells. Eur. J. Nutr. 56, 1077–1084. 10.1007/s00394-016-1157-5 26830415

[B28] Chen Y. Song M. Riley J. P. Hu C. C. Peng X. Scheuner D.. (2016). A selective GPR40 (FFAR1) agonist LY2881835 provides immediate and durable glucose control in rodent models of type 2 diabetes. Pharmacol. Res. Perspect. 4:e00278. 10.1002/prp2.278 28097011 PMC5226292

[B29] Cornish J. MacGibbon A. Lin J. M. Watson M. Callon K. E. Tong P. C.. (2008). Modulation of osteoclastogenesis by fatty acids. Endocrinology 149, 5688–5695. 10.1210/en.2008-0111 18617622

[B30] Cucchi D. Camacho-Munoz D. Certo M. Niven J. Smith J. Nicolaou A.. (2020). Omega-3 polyunsaturated fatty acids impinge on CD4+ T cell motility and adipose tissue distribution via direct and lipid mediator-dependent effects. Cardiovasc. Res. 116, 1006–1020. 10.1093/cvr/cvz208 31399738

[B31] Cury-Boaventura M. F. Pompeia C. Curi R. (2004). Comparative toxicity of oleic acid and linoleic acid on Jurkat cells. Clin. Nutr. 23, 721–732. 10.1016/j.clnu.2003.12.004 15297111

[B32] Davanso M. R. Crisma A. R. Braga T. T. Masi L. N. do Amaral C. L. Leal V. N. C.. (2021). Macrophage inflammatory state in Type 1 diabetes: triggered by NLRP3/iNOS pathway and attenuated by docosahexaenoic acid. Clin. Sci. 135, 19–34. 10.1042/CS20201348 33399849

[B33] de Jong A. J. Kloppenburg M. Toes R. E. Ioan-Facsinay A. (2014). Fatty acids, lipid mediators, and T-cell function. Front. Immunol. 5, 483. 10.3389/fimmu.2014.00483 25352844 PMC4195378

[B34] de Lima T. M. de Sa Lima L. Scavone C. Curi R. (2006). Fatty acid control of nitric oxide production by macrophages. FEBS Lett. 580, 3287–3295. 10.1016/j.febslet.2006.04.091 16698019

[B35] Denys A. Hichami A. Khan N. A. (2005). n-3 PUFAs modulate T-cell activation via protein kinase C-alpha and -epsilon and the NF-kappaB signaling pathway. J. Lipid Res. 46, 752–758. 10.1194/jlr.M400444-JLR200 15627650

[B36] Egerod K. L. Engelstoft M. S. Lund M. L. Grunddal K. V. Zhao M. Barir-Jensen D.. (2015). Transcriptional and functional characterization of the G protein-coupled receptor repertoire of gastric somatostatin cells. Endocrinology 156, 3909–3923. 10.1210/EN.2015-1388 26181106

[B37] Engelstoft M. S. Park W. M. Sakata I. Kristensen L. V. Husted A. S. Osborne-Lawrence S.. (2013). Seven transmembrane G protein-coupled receptor repertoire of gastric ghrelin cells. Mol. Metab. 2, 376–392. 10.1016/j.molmet.2013.08.006 24327954 PMC3854997

[B38] Falagas M. E. Kompoti M. (2006). Obesity and infection. Lancet Infect. Dis. 6, 438–446. 10.1016/S1473-3099(06)70523-0 16790384

[B39] Farjadian S. Moghtaderi M. Kalani M. Gholami T. Hosseini Teshnizi S. (2016). Effects of omega-3 fatty acids on serum levels of T-helper cytokines in children with asthma. Cytokine 85, 61–66. 10.1016/j.cyto.2016.06.002 27288633

[B40] Fickl H. Cockeran R. Steel H. C. Feldman C. Cowan G. Mitchell T. J.. (2005). Pneumolysin-mediated activation of NFkappaB in human neutrophils is antagonized by docosahexaenoic acid. Clin. Exp. Immunol. 140, 274–281. 10.1111/j.1365-2249.2005.02757.x 15807851 PMC1809376

[B41] Fowler K. H. Chapkin R. S. McMurray D. N. (1993). Effects of purified dietary n-3 ethyl esters on murine T lymphocyte function. J. Immunol. 151, 5186–5197. 8228217

[B42] Fritsche K. L. Byrge M. Feng C. (1999). Dietary omega-3 polyunsaturated fatty acids from fish oil reduce interleukin-12 and interferon-gamma production in mice. Immunol. Lett. 65, 167–173. 10.1016/S0165-2478(98)00109-6 10065739

[B43] Fu H. Tang B. Lang J. Du Y. Cao B. Jin L.. (2020). High-fat diet promotes macrophage-mediated hepatic inflammation and aggravates diethylnitrosamine-induced hepatocarcinogenesis in mice. Front. Nutr. 7:585306. 10.3389/fnut.2020.585306 33304918 PMC7701255

[B44] Gani O. A. Sylte I. (2008). Molecular recognition of docosahexaenoic acid by peroxisome proliferator-activated receptors and retinoid-X receptor alpha. J. Mol. Graph. Model. 27, 217–224. 10.1016/j.jmgm.2008.04.008 18547851

[B45] Gorjao R. Cury-Boaventura M. F. de Lima T. M. Curi R. (2007). Regulation of human lymphocyte proliferation by fatty acids. Cell Biochem. Funct. 25, 305–315. 10.1002/cbf.1388 17195961

[B46] Gorjao R. Verlengia R. Lima T. M. Soriano F. G. Boaventura M. F. Kanunfre C. C.. (2006). Effect of docosahexaenoic acid-rich fish oil supplementation on human leukocyte function. Clin. Nutr. 25, 923–938. 10.1016/j.clnu.2006.03.004 16697494

[B47] Gozal D. Qiao Z. Almendros I. Zheng J. Khalyfa A. Shimpukade B.. (2016). Treatment with TUG891, a free fatty acid receptor 4 agonist, restores adipose tissue metabolic dysfunction following chronic sleep fragmentation in mice. Int. J. Obes. 40, 1143–1149. 10.1038/ijo.2016.37 26980479

[B48] Gregersen P. K. Behrens T. W. (2006). Genetics of autoimmune diseases–disorders of immune homeostasis. Nat. Rev. Genet. 7, 917–928. 10.1038/nrg1944 17139323

[B49] Hara T. Hirasawa A. Sun Q. Sadakane K. Itsubo C. Iga T.. (2009). Novel selective ligands for free fatty acid receptors GPR120 and GPR40. Naunyn Schmiedebergs. Arch. Pharmacol. 380, 247–255. 10.1007/s00210-009-0425-9 19471906

[B50] Harmon D. B. Wu C. Dedousis N. Sipula I. J. Stefanovic-Racic M. Schoiswohl G.. (2018). Adipose tissue-derived free fatty acids initiate myeloid cell accumulation in mouse liver in states of lipid oversupply. Am. J. Physiol. Endocrinol. Metab. 315, E758–E770. 10.1152/ajpendo.00172.2018 30086648 PMC6293173

[B51] Hasan A. U. Ohmori K. Hashimoto T. Kamitori K. Yamaguchi F. Noma T.. (2017). GPR120 in adipocytes has differential roles in the production of pro-inflammatory adipocytokines. Biochem. Biophys. Res. Commun. 486, 76–82. 10.1016/j.bbrc.2017.03.001 28263744

[B52] Hatanaka E. Levada-Pires A. C. Pithon-Curi T. C. Curi R. (2006). Systematic study on ROS production induced by oleic, linoleic, and gamma-linolenic acids in human and rat neutrophils. Free Radic. Biol. Med. 41, 1124–1132. 10.1016/j.freeradbiomed.2006.06.014 16962937

[B53] Hauge M. Vestmar M. A. Husted A. S. Ekberg J. P. Wright M. J. Di Salvo J.. (2015). GPR40 (FFAR1) - combined Gs and Gq signaling *in vitro* is associated with robust incretin secretagogue action *ex vivo* and *in vivo*. Mol. Metab. 4, 3–14. 10.1016/j.molmet.2014.10.002 25685685 PMC4314522

[B54] Hidalgo M. A. Nahuelpan C. Manosalva C. Jara E. Carretta M. D. Conejeros I.. (2011). Oleic acid induces intracellular calcium mobilization, MAPK phosphorylation, superoxide production and granule release in bovine neutrophils. Biochem. Biophys. Res. Commun. 409, 280–286. 10.1016/j.bbrc.2011.04.144 21575602

[B55] Hirasawa A. Tsumaya K. Awaji T. Katsuma S. Adachi T. Yamada M.. (2005). Free fatty acids regulate gut incretin glucagon-like peptide-1 secretion through GPR120. Nat. Med. 11, 90–94. 10.1038/nm1168 15619630

[B56] Houze J. B. Zhu L. Sun Y. Akerman M. Qiu W. Zhang A. J.. (2012). AMG 837: a potent, orally bioavailable GPR40 agonist. Bioorg. Med. Chem. Lett. 22, 1267–1270. 10.1016/j.bmcl.2011.10.118 22217876

[B57] Hu H. He L. Y. Gong Z. Li N. Lu Y. N. Zhai Q. W.. (2009). A novel class of antagonists for the FFAs receptor GPR40. Biochem. Biophys. Res. Commun. 390, 557–563. 10.1016/j.bbrc.2009.10.004 19818732

[B58] Hu X. Zhou J. Song S. S. Kong W. Shi Y. C. Chen L. L.. (2020). TLR4/AP-1-targeted anti-inflammatory intervention attenuates insulin sensitivity and liver steatosis. Mediators Inflamm. 2020:2960517. 10.1155/2020/2960517 33013197 PMC7519185

[B59] Huang S. Rutkowsky J. M. Snodgrass R. G. Ono-Moore K. D. Schneider D. A. Newman J. W.. (2012). Saturated fatty acids activate TLR-mediated proinflammatory signaling pathways. J. Lipid Res. 53, 2002–2013. 10.1194/jlr.D029546 22766885 PMC3413240

[B60] Huang X. Yi S. Hu J. Du Z. Wang Q. Ye Z.. (2020). Linoleic acid inhibits *in vitro* function of human and murine dendritic cells, CD4(+)T cells and retinal pigment epithelial cells. Graefes Arch. Clin. Exp. Ophthalmol. 259, 987–998. 10.1007/s00417-020-04972-6 33079282

[B61] Huang Z. H. Gu D. Mazzone T. (2004). Oleic acid modulates the post-translational glycosylation of macrophage ApoE to increase its secretion. J. Biol. Chem. 279, 29195–29201. 10.1074/jbc.M402631200 15131109

[B62] Hudson B. D. Shimpukade B. Mackenzie A. E. Butcher A. J. Pediani J. D. Christiansen E.. (2013). The pharmacology of TUG-891, a potent and selective agonist of the free fatty acid receptor 4 (FFA4/GPR120), demonstrates both potential opportunity and possible challenges to therapeutic agonism. Mol. Pharmacol. 84, 710–725. 10.1124/mol.113.087783 23979972 PMC3807074

[B63] Im D. S. (2016). Functions of omega-3 fatty acids and FFA4 (GPR120) in macrophages. Eur. J. Pharmacol. 785, 36–43. 10.1016/j.ejphar.2015.03.094 25987421

[B64] Innes J. K. Calder P. C. (2018). Omega-6 fatty acids and inflammation. Prostaglandins Leukot. Essent. Fatty Acids 132, 41–48. 10.1016/j.plefa.2018.03.004 29610056

[B65] Ioan-Facsinay A. Kwekkeboom J. C. Westhoff S. Giera M. Rombouts Y. van Harmelen V.. (2013). Adipocyte-derived lipids modulate CD4+ T-cell function. Eur. J. Immunol. 43, 1578–1587. 10.1002/eji.201243096 23504601

[B66] Itoh Y. Kawamata Y. Harada M. Kobayashi M. Fujii R. Fukusumi S.. (2003). Free fatty acids regulate insulin secretion from pancreatic beta cells through GPR40. Nature 422, 173–176. 10.1038/nature01478 12629551

[B67] Jaudszus A. Gruen M. Watzl B. Ness C. Roth A. Lochner A.. (2013). Evaluation of suppressive and pro-resolving effects of EPA and DHA in human primary monocytes and T-helper cells. J. Lipid Res. 54, 923–935. 10.1194/jlr.P031260 23349208 PMC3605999

[B68] Jeffery N. M. Newsholme E. A. Calder P. C. (1997). Level of polyunsaturated fatty acids and the n-6 to n-3 polyunsaturated fatty acid ratio in the rat diet alter serum lipid levels and lymphocyte functions. Prostaglandins Leukot. Essent. Fatty Acids 57, 149–160. 10.1016/S0952-3278(97)90005-3 9250698

[B69] Jeffery N. M. Sanderson P. Sherrington E. J. Newsholme E. A. Calder P. C. (1996). The ratio of n-6 to n-3 polyunsaturated fatty acids in the rat diet alters serum lipid levels and lymphocyte functions. Lipids 31, 737–745. 10.1007/BF02522890 8827697

[B70] Jia Y. C. Qiu S. Xu J. Kang Q. L. Chai Y. M. (2020). Docosahexaenoic acid improves diabetic wound healing in a rat model by restoring impaired plasticity of macrophage progenitor cells. Plast Reconstr. Surg. 145, 942e−950e. 10.1097/PRS.0000000000006739 32332536

[B71] Kain V. Halade G. V. (2019). Immune responsive resolvin D1 programs peritoneal macrophages and cardiac fibroblast phenotypes in diversified metabolic microenvironment. J. Cell. Physiol. 234, 3910–3920. 10.1002/jcp.27165 30191990 PMC6317995

[B72] Kaku K. Enya K. Nakaya R. Ohira T. Matsuno R. (2015). Efficacy and safety of fasiglifam (TAK-875), a G protein-coupled receptor 40 agonist, in Japanese patients with type 2 diabetes inadequately controlled by diet and exercise: a randomized, double-blind, placebo-controlled, phase III trial. Diabetes Obes. Metab. 17, 675–681. 10.1111/dom.12467 25787200 PMC4676912

[B73] Kang S. Huang J. Lee B. K. Jung Y. S. Im E. Koh J. M.. (2018). Omega-3 polyunsaturated fatty acids protect human hepatoma cells from developing steatosis through FFA4 (GPR120). Biochim. Biophys. Acta Mol. Cell. Biol. Lipids 1863, 105–116. 10.1016/j.bbalip.2017.11.002 29126901

[B74] Karasawa T. Kawashima A. Usui-Kawanishi F. Watanabe S. Kimura H. Kamata R.. (2018). Saturated fatty acids undergo intracellular crystallization and activate the NLRP3 inflammasome in macrophages. Arterioscler. Thromb. Vasc. Biol. 38, 744–756. 10.1161/ATVBAHA.117.310581 29437575

[B75] Karsten S. Schafer G. Schauder P. (1994). Cytokine production and DNA synthesis by human peripheral lymphocytes in response to palmitic, stearic, oleic, and linoleic acid. J. Cell. Physiol. 161, 15–22. 10.1002/jcp.1041610103 7929601

[B76] Kasonga A. E. Kruger M. C. Coetzee M. (2019). Free fatty acid receptor 4-beta-arrestin 2 pathway mediates the effects of different classes of unsaturated fatty acids in osteoclasts and osteoblasts. Biochim. Biophys. Acta Mol. Cell. Biol. Lipids 1864, 281–289. 10.1016/j.bbalip.2018.12.009 30578965

[B77] Kawano A. Ariyoshi W. Yoshioka Y. Hikiji H. Nishihara T. Okinaga T. (2019). Docosahexaenoic acid enhances M2 macrophage polarization via the p38 signaling pathway and autophagy. J. Cell. Biochem. 120, 12604–12617. 10.1002/jcb.28527 30825233

[B78] Khan M. A. Pace-Asciak C. Al-Hassan J. M. Afzal M. Liu Y. F. Oommen S.. (2018). Furanoid F-acid F6 uniquely induces NETosis compared to C16 and C18 fatty acids in human neutrophils. Biomolecules 8:144. 10.3390/biom8040144 30428625 PMC6315434

[B79] Kim D. H. Cho Y. M. Lee K. H. Jeong S. W. Kwon O. J. (2017). Oleate protects macrophages from palmitate-induced apoptosis through the downregulation of CD36 expression. Biochem. Biophys. Res. Commun. 488, 477–482. 10.1016/j.bbrc.2017.05.066 28522296

[B80] Kim S. M. Park E. J. Kim J. Y. Choi J. Lee H. J. (2020). Anti-inflammatory effects of fermented lotus root and linoleic acid in lipopolysaccharide-induced RAW 264.7 cells. Life 10:293. 10.3390/life10110293 33228085 PMC7699317

[B81] Kimura I. Ichimura A. Ohue-Kitano R. Igarashi M. (2020). Free fatty acid receptors in health and disease. Physiol. Rev. 100, 171–210. 10.1152/physrev.00041.2018 31487233

[B82] Konda V. R. Desai A. Darland G. Grayson N. Bland J. S. (2014). KDT501, a derivative from hops, normalizes glucose metabolism and body weight in rodent models of diabetes. PLoS ONE 9:e87848. 10.1371/journal.pone.0087848 24498211 PMC3907559

[B83] Korbecki J. Bajdak-Rusinek K. (2019). The effect of palmitic acid on inflammatory response in macrophages: an overview of molecular mechanisms. Inflamm. Res. 68, 915–932. 10.1007/s00011-019-01273-5 31363792 PMC6813288

[B84] Kotarsky K. Nilsson N. E. Flodgren E. Owman C. Olde B. (2003). A human cell surface receptor activated by free fatty acids and thiazolidinedione drugs. Biochem. Biophys. Res. Commun. 301, 406–410. 10.1016/S0006-291X(02)03064-4 12565875

[B85] Kris-Etherton P. M. (1999). AHA science advisory. Monounsaturated fatty acids and risk of cardiovascular disease. American Heart Association. Nutrition Committee. Circulation 100, 1253–1258. 10.1161/01.CIR.100.11.1253 10484550

[B86] Kristinsson H. Smith D. M. Bergsten P. Sargsyan E. (2013). FFAR1 is involved in both the acute and chronic effects of palmitate on insulin secretion. Endocrinology 154, 4078–4088. 10.1210/en.2013-1352 24035997

[B87] Lager S. Ramirez V. I. Gaccioli F. Jansson T. Powell T. L. (2014). Expression and localization of the omega-3 fatty acid receptor GPR120 in human term placenta. Placenta 35, 523–525. 10.1016/j.placenta.2014.04.017 24844436 PMC4096656

[B88] Lamas Bervejillo M. Bonanata J. Franchini G. R. Richeri A. Marques J. M. Freeman B. A.. (2020). A FABP4-PPARgamma signaling axis regulates human monocyte responses to electrophilic fatty acid nitroalkenes. Redox Biol. 29:101376. 10.1016/j.redox.2019.101376 31926616 PMC6926352

[B89] Lancaster G. I. Langley K. G. Berglund N. A. Kammoun H. L. Reibe S. Estevez E.. (2018). Evidence that TLR4 is not a receptor for saturated fatty acids but mediates lipid-induced inflammation by reprogramming macrophage metabolism. Cell Metab. 27, 1096–1110 e1095. 10.1016/j.cmet.2018.03.014 29681442

[B90] Lee J. Y. Plakidas A. Lee W. H. Heikkinen A. Chanmugam P. Bray G.. (2003). Differential modulation of Toll-like receptors by fatty acids: preferential inhibition by n-3 polyunsaturated fatty acids. J. Lipid Res. 44, 479–486. 10.1194/jlr.M200361-JLR200 12562875

[B91] LeMieux M. J. Kalupahana N. S. Scoggin S. Moustaid-Moussa N. (2015). Eicosapentaenoic acid reduces adipocyte hypertrophy and inflammation in diet-induced obese mice in an adiposity-independent manner. J. Nutr. 145, 411–417. 10.3945/jn.114.202952 25733455

[B92] Li X. Bi X. Wang S. Zhang Z. Li F. Zhao A. Z. (2019). Therapeutic potential of omega-3 polyunsaturated fatty acids in human autoimmune diseases. Front. Immunol. 10:2241. 10.3389/fimmu.2019.02241 31611873 PMC6776881

[B93] Li X. Yu Y. Funk C. D. (2013). Cyclooxygenase-2 induction in macrophages is modulated by docosahexaenoic acid via interactions with free fatty acid receptor 4 (FFA4). FASEB J. 27, 4987–4997. 10.1096/fj.13-235333 24005906

[B94] Liang P. Henning S. M. Guan J. Grogan T. Elashoff D. Cohen P.. (2020). Effect of dietary omega-3 fatty acids on castrate-resistant prostate cancer and tumor-associated macrophages. Prostate Cancer Prostatic Dis. 23, 127–135. 10.1038/s41391-019-0168-8 31439889 PMC7031053

[B95] Lima T. M. Kanunfre C. C. Pompeia C. Verlengia R. Curi R. (2002). Ranking the toxicity of fatty acids on Jurkat and Raji cells by flow cytometric analysis. Toxicol. Vitro 16, 741–747. 10.1016/S0887-2333(02)00095-4 12423658

[B96] Lin D. C. Guo Q. Luo J. Zhang J. Nguyen K. Chen M.. (2012). Identification and pharmacological characterization of multiple allosteric binding sites on the free fatty acid 1 receptor. Mol. Pharmacol. 82, 843–859. 10.1124/mol.112.079640 22859723 PMC3477236

[B97] Liu Y. Chen L. Y. Sokolowska M. Eberlein M. Alsaaty S. Martinez-Anton A.. (2014). The fish oil ingredient, docosahexaenoic acid, activates cytosolic phospholipase A(2) via GPR120 receptor to produce prostaglandin E(2) and plays an anti-inflammatory role in macrophages. Immunology 143, 81–95. 10.1111/imm.12296 24673159 PMC4137958

[B98] Loaiza A. Carretta M. D. Taubert A. Hermosilla C. Hidalgo M. A. Burgos R. A. (2016). Differential intracellular calcium influx, nitric oxide production, ICAM-1 and IL8 expression in primary bovine endothelial cells exposed to nonesterified fatty acids. BMC Vet. Res. 12:38. 10.1186/s12917-016-0654-3 26916791 PMC4766702

[B99] Lu Z. Li Y. Brinson C. W. Kirkwood K. L. Lopes-Virella M. F. Huang Y. (2017). CD36 is upregulated in mice with periodontitis and metabolic syndrome and involved in macrophage gene upregulation by palmitate. Oral Dis. 23, 210–218. 10.1111/odi.12596 27753178 PMC5303180

[B100] Luo J. Swaminath G. Brown S. P. Zhang J. Guo Q. Chen M.. (2012). A potent class of GPR40 full agonists engages the enteroinsular axis to promote glucose control in rodents. PLoS ONE 7:e46300. 10.1371/journal.pone.0046300 23056280 PMC3467217

[B101] Ma C. Kesarwala A. H. Eggert T. Medina-Echeverz J. Kleiner D. E. Jin P.. (2016). NAFLD causes selective CD4(+) T lymphocyte loss and promotes hepatocarcinogenesis. Nature 531, 253–257. 10.1038/nature16969 26934227 PMC4786464

[B102] Mabalirajan U. Rehman R. Ahmad T. Kumar S. Singh S. Leishangthem G. D.. (2013). Linoleic acid metabolite drives severe asthma by causing airway epithelial injury. Sci. Rep. 3:1349. 10.1038/srep01349 23443229 PMC3583002

[B103] Magdalon J. Vinolo M. A. Rodrigues H. G. Paschoal V. A. Torres R. P. Mancini-Filho J.. (2012). Oral administration of oleic or linoleic acids modulates the production of inflammatory mediators by rat macrophages. Lipids 47, 803–812. 10.1007/s11745-012-3687-9 22695743

[B104] Mancini A. D. Bertrand G. Vivot K. Carpentier E. Tremblay C. Ghislain J.. (2015). beta-arrestin recruitment and biased agonism at free fatty acid receptor 1. J. Biol. Chem. 290, 21131–21140. 10.1074/jbc.M115.644450 26157145 PMC4543669

[B105] Manosalva C. Mena J. Velasquez Z. Colenso C. K. Brauchi S. Burgos R. A.. (2015). Cloning, identification and functional characterization of bovine free fatty acid receptor-1 (FFAR1/GPR40) in neutrophils. PLoS ONE 10:e0119715. 10.1371/journal.pone.0119715 25790461 PMC4366208

[B106] Mantovani A. Cassatella M. A. Costantini C. Jaillon S. (2011). Neutrophils in the activation and regulation of innate and adaptive immunity. Nat. Rev. Immunol. 11, 519–531. 10.1038/nri3024 21785456

[B107] Mathers A. R. Carey C. D. Killeen M. E. Diaz-Perez J. A. Salvatore S. R. Schopfer F. J.. (2017). Electrophilic nitro-fatty acids suppress allergic contact dermatitis in mice. Allergy 72, 656–664. 10.1111/all.13067 27718238 PMC5352476

[B108] McDaniel J. C. Szalacha L. Sales M. Roy S. Chafee S. Parinandi N. (2017). EPA + DHA supplementation reduces PMN activation in microenvironment of chronic venous leg ulcers: a randomized, double-blind, controlled study. Wound Repair Regen. 25, 680–690. 10.1111/wrr.12558 28758717 PMC9288800

[B109] Medeiros-de-Moraes I. M. Goncalves-de-Albuquerque C. F. Kurz A. R. M. Oliveira F. M. J. de Abreu V. H. P. Torres R. C.. (2018). Omega-9 oleic acid, the main compound of olive oil, mitigates inflammation during experimental sepsis. Oxid. Med. Cell. Longev. 2018:6053492. 10.1155/2018/6053492 30538802 PMC6260523

[B110] Mena S. J. Manosalva C. Carretta M. D. Teuber S. Olmo I. Burgos R. A.. (2016). Differential free fatty acid receptor-1 (FFAR1/GPR40) signalling is associated with gene expression or gelatinase granule release in bovine neutrophils. Innate Immun. 22, 479–489. 10.1177/1753425916656765 27363707

[B111] Menon V. Lincoff A. M. Nicholls S. J. Jasper S. Wolski K. McGuire D. K.. (2018). Fasiglifam-induced liver injury in patients with type 2 diabetes: results of a randomized controlled cardiovascular outcomes safety trial. Diabetes Care 41, 2603–2609. 10.2337/dc18-0755 30459247

[B112] Meydani S. N. Endres S. Woods M. M. Goldin B. R. Soo C. Morrill-Labrode A.. (1991). Oral (n-3) fatty acid supplementation suppresses cytokine production and lymphocyte proliferation: comparison between young and older women. J. Nutr. 121, 547–555. 10.1093/jn/121.4.547 2007907

[B113] Milligan G. Shimpukade B. Ulven T. Hudson B. D. (2017). Complex pharmacology of free fatty acid receptors. Chem. Rev. 117, 67–110. 10.1021/acs.chemrev.6b00056 27299848

[B114] Mohammad S. (2016). GPR40 agonists for the treatment of type 2 diabetes mellitus: benefits and challenges. Curr. Drug Targets 17, 1292–1300. 10.2174/1389450117666151209122702 26648068

[B115] Muller A. K. Schmolz L. Wallert M. Schubert M. Schlormann W. Glei M.. (2019). *In vitro* digested nut oils attenuate the lipopolysaccharide-induced inflammatory response in macrophages. Nutrients 11:503. 10.3390/nu11030503 30818812 PMC6471109

[B116] Negoro N. Sasaki S. Mikami S. Ito M. Suzuki M. Tsujihata Y.. (2010). Discovery of TAK-875: a potent, selective, and orally bioavailable GPR40 agonist. ACS Med. Chem. Lett. 1, 290–294. 10.1021/ml1000855 24900210 PMC4007909

[B117] Oh D. Y. Talukdar S. Bae E. J. Imamura T. Morinaga H. Fan W.. (2010). GPR120 is an omega-3 fatty acid receptor mediating potent anti-inflammatory and insulin-sensitizing effects. Cell 142, 687–698. 10.1016/j.cell.2010.07.041 20813258 PMC2956412

[B118] Oh D. Y. Walenta E. Akiyama T. E. Lagakos W. S. Lackey D. Pessentheiner A. R.. (2014). A Gpr120-selective agonist improves insulin resistance and chronic inflammation in obese mice. Nat. Med. 20, 942–947. 10.1038/nm.3614 24997608 PMC4126875

[B119] Olmo I. Teuber S. Larrazabal C. Alarcon P. Raipane F. Burgos R. A.. (2019). Docosahexaenoic acid and TUG-891 activate free fatty acid-4 receptor in bovine neutrophils. Vet. Immunol. Immunopathol. 209, 53–60. 10.1016/j.vetimm.2019.02.008 30885306

[B120] Otton R. Curi R. (2005). Toxicity of a mixture of fatty acids on human blood lymphocytes and leukaemia cell lines. Toxicol. Vitro 19, 749–755. 10.1016/j.tiv.2005.04.003 15908173

[B121] Panda L. Gheware A. Rehman R. Yadav M. K. Jayaraj B. S. Madhunapantula S. V.. (2017). Linoleic acid metabolite leads to steroid resistant asthma features partially through NF-kappaB. Sci. Rep. 7:9565. 10.1038/s41598-017-09869-9 28851976 PMC5575291

[B122] Paschoal V. A. Vinolo M. A. Crisma A. R. Magdalon J. Curi R. (2013). Eicosapentaenoic (EPA) and docosahexaenoic (DHA) acid differentially modulate rat neutrophil function *in vitro*. Lipids 48, 93–103. 10.1007/s11745-012-3726-6 23086551

[B123] Pereira L. M. Hatanaka E. Martins E. F. Oliveira F. Liberti E. A. Farsky S. H.. (2008). Effect of oleic and linoleic acids on the inflammatory phase of wound healing in rats. Cell Biochem. Funct. 26, 197–204. 10.1002/cbf.1432 17918246

[B124] Perez-Mojica J. E. Lillycrop K. A. Cooper C. Calder P. C. Burdge G. C. (2020). Docosahexaenoic acid and oleic acid induce altered DNA methylation of individual CpG loci in Jurkat T cells. Prostaglandins Leukot. Essent. Fatty Acids 158:102128. 10.1016/j.plefa.2020.102128 32464433

[B125] Peterson L. D. Jeffery N. M. Thies F. Sanderson P. Newsholme E. A. Calder P. C. (1998). Eicosapentaenoic and docosahexaenoic acids alter rat spleen leukocyte fatty acid composition and prostaglandin E2 production but have different effects on lymphocyte functions and cell-mediated immunity. Lipids 33, 171–180. 10.1007/s11745-998-0193-y 9507239

[B126] Pham T. X. Bae M. Lee Y. Park Y. K. Lee J. Y. (2018). Transcriptional and posttranscriptional repression of histone deacetylases by docosahexaenoic acid in macrophages. J. Nutr. Biochem. 57, 162–169. 10.1016/j.jnutbio.2018.03.002 29734115

[B127] Pisani L. F. Lecchi C. Invernizzi G. Sartorelli P. Savoini G. Ceciliani F. (2009). *In vitro* modulatory effect of omega-3 polyunsaturated fatty acid (EPA and DHA) on phagocytosis and ROS production of goat neutrophils. Vet. Immunol. Immunopathol. 131, 79–85. 10.1016/j.vetimm.2009.03.018 19395090

[B128] Pompura S. L. Wagner A. Kitz A. Laperche J. Yosef N. Dominguez-Villar M.. (2020). Oleic acid restores suppressive defects in tissue-resident FOXP3 regulatory T cells from patients with multiple sclerosis. *J. Clin*. Invest. 131:e138519. 10.1172/JCI138519 PMC7810477 33170805

[B129] Prescott J. Owens D. Collins P. Johnson A. Tomkin G. H. (1999). The fatty acid distribution in low density lipoprotein in diabetes. Biochim. Biophys. Acta 1439, 110–116. 10.1016/S1388-1981(99)00082-7 10395970

[B130] Rahman M. M. Bhattacharya A. Fernandes G. (2006). Conjugated linoleic acid inhibits osteoclast differentiation of RAW264.7 cells by modulating RANKL signaling. J. Lipid Res. 47, 1739–1748. 10.1194/jlr.M600151-JLR200 16702601

[B131] Rahman M. M. Bhattacharya A. Fernandes G. (2008). Docosahexaenoic acid is more potent inhibitor of osteoclast differentiation in RAW 264.7 cells than eicosapentaenoic acid. J. Cell. Physiol. 214, 201–209. 10.1002/jcp.21188 17929247

[B132] Reddy A. T. Lakshmi S. P. Dornadula S. Pinni S. Rampa D. R. Reddy R. C. (2013). The nitrated fatty acid 10-nitro-oleate attenuates allergic airway disease. J. Immunol. 191, 2053–2063. 10.4049/jimmunol.1300730 23913958

[B133] Reyes-Quiroz M. E. Alba G. Saenz J. Santa-Maria C. Geniz I. Jimenez J.. (2014). Oleic acid modulates mRNA expression of liver X receptor (LXR) and its target genes ABCA1 and SREBP1c in human neutrophils. Eur. J. Nutr. 53, 1707–1717. 10.1007/s00394-014-0677-0 24722912

[B134] Riediger N. D. Othman R. A. Suh M. Moghadasian M. H. (2009). A systemic review of the roles of n-3 fatty acids in health and disease. J. Am. Diet. Assoc. 109, 668–679. 10.1016/j.jada.2008.12.022 19328262

[B135] Rodrigues H. G. Takeo Sato F. Curi R. Vinolo M. A. R. (2016). Fatty acids as modulators of neutrophil recruitment, function and survival. Eur. J. Pharmacol. 785, 50–58. 10.1016/j.ejphar.2015.03.098 25987417

[B136] Rodrigues H. G. Vinolo M. A. Magdalon J. Fujiwara H. Cavalcanti D. M. Farsky S. H.. (2010). Dietary free oleic and linoleic acid enhances neutrophil function and modulates the inflammatory response in rats. Lipids 45, 809–819. 10.1007/s11745-010-3461-9 20730605

[B137] Rohrig F. Schulze A. (2016). The multifaceted roles of fatty acid synthesis in cancer. Nat. Rev. Cancer 16, 732–749. 10.1038/nrc.2016.89 27658529

[B138] Rudolph V. Rudolph T. K. Schopfer F. J. Bonacci G. Woodcock S. R. Cole M. P.. (2010). Endogenous generation and protective effects of nitro-fatty acids in a murine model of focal cardiac ischaemia and reperfusion. Cardiovasc. Res. 85, 155–166. 10.1093/cvr/cvp275 19666678 PMC2791055

[B139] Rybicka M. Stachowska E. Gutowska I. Parczewski M. Baskiewicz M. Machalinski B.. (2011). Comparative effects of conjugated linoleic acid (CLA) and linoleic acid (LA) on the oxidoreduction status in THP-1 macrophages. J. Agric. Food Chem. 59, 4095–4103. 10.1021/jf103647n 21391598

[B140] Saidi H. Murtaza B. Khan A. S. Koceir E. A. Hichami A. Khan N. A. (2020). DHA induces Jurkat T-cell arrest in G2/M phase of cell cycle and modulates the plasma membrane expression of TRPC3/6 channels. Biochimie 181, 169–175. 10.1016/j.biochi.2020.12.005 33333171

[B141] Saini S. Rai A. K. (2020). Linoleic acid inhibits the release of leishmania donovani derived microvesicles and decreases its survival in macrophages. Front. Cell. Infect. Microbiol. 10:406. 10.3389/fcimb.2020.00406 32850500 PMC7426612

[B142] Sano M. Shimazaki S. Kaneko Y. Karasawa T. Takahashi M. Ohkuchi A.. (2020). Palmitic acid activates NLRP3 inflammasome and induces placental inflammation during pregnancy in mice. J. Reprod. Dev. 66, 241–248. 10.1262/jrd.2020-007 32101829 PMC7297640

[B143] Sawzdargo M. George S. R. Nguyen T. Xu S. Kolakowski L. F. O'Dowd B. F. (1997). A cluster of four novel human G protein-coupled receptor genes occurring in close proximity to CD22 gene on chromosome 19q13.1. Biochem. Biophys. Res. Commun. 239, 543–547. 10.1006/bbrc.1997.7513 9344866

[B144] Serhan C. N. (2014). Pro-resolving lipid mediators are leads for resolution physiology. Nature 510, 92–101. 10.1038/nature13479 24899309 PMC4263681

[B145] Sheridan P. A. Paich H. A. Handy J. Karlsson E. A. Hudgens M. G. Sammon A. B.. (2012). Obesity is associated with impaired immune response to influenza vaccination in humans. Int. J. Obes. 36, 1072–1077. 10.1038/ijo.2011.208 22024641 PMC3270113

[B146] Sierra S. Lara-Villoslada F. Comalada M. Olivares M. Xaus J. (2008). Dietary eicosapentaenoic acid and docosahexaenoic acid equally incorporate as decosahexaenoic acid but differ in inflammatory effects. Nutrition 24, 245–254. 10.1016/j.nut.2007.11.005 18312787

[B147] Silva-Martinez G. A. Rodriguez-Rios D. Alvarado-Caudillo Y. Vaquero A. Esteller M. Carmona F. J.. (2016). Arachidonic and oleic acid exert distinct effects on the DNA methylome. Epigenetics 11, 321–334. 10.1080/15592294.2016.1161873 27088456 PMC4889238

[B148] Simopoulos A. P. (2008). The importance of the omega-6/omega-3 fatty acid ratio in cardiovascular disease and other chronic diseases. Exp. Biol. Med. 233, 674–688. 10.3181/0711-MR-311 18408140

[B149] Son S. E. Kim N. J. Im D. S. (2021). Development of free fatty acid receptor 4 (FFA4/GPR120) agonists in health science. Biomol. Ther. 29, 22–30. 10.4062/biomolther.2020.213 33372166 PMC7771848

[B150] Son S. E. Park S. J. Koh J. M. Im D. S. (2020). Free fatty acid receptor 4 (FFA4) activation ameliorates 2,4-dinitrochlorobenzene-induced atopic dermatitis by increasing regulatory T cells in mice. Acta Pharmacol. Sin. 41, 1337–1347. 10.1038/s41401-020-0435-1 32555509 PMC7609340

[B151] Souza P. R. Walker M. E. Goulding N. J. Dalli J. Perretti M. Norling L. V. (2020). The GPR40 agonist GW9508 enhances neutrophil function to aid bacterial clearance during *E. coli* infections. Front. Immunol. 11:573019. 10.3389/fimmu.2020.573019 33133087 PMC7550532

[B152] Sparks S. M. Chen G. Collins J. L. Danger D. Dock S. T. Jayawickreme C.. (2014). Identification of diarylsulfonamides as agonists of the free fatty acid receptor 4 (FFA4/GPR120). Bioorg. Med. Chem. Lett. 24, 3100–3103. 10.1016/j.bmcl.2014.05.012 24881566

[B153] Stachowska E. Kijowski J. Dziedziejko V. Siennicka A. Chlubek D. (2011). Conjugated linoleic acid regulates phosphorylation of PPARgamma by modulation of ERK 1/2 and p38 signaling in human macrophages/fatty acid-laden macrophages. J. Agric. Food Chem. 59, 11846–11852. 10.1021/jf2014233 21854054

[B154] Stelzner K. Herbert D. Popkova Y. Lorz A. Schiller J. Gericke M.. (2016). Free fatty acids sensitize dendritic cells to amplify TH1/TH17-immune responses. Eur. J. Immunol. 46, 2043–2053. 10.1002/eji.201546263 27214608

[B155] Stentz F. B. Kitabchi A. E. (2006). Palmitic acid-induced activation of human T-lymphocytes and aortic endothelial cells with production of insulin receptors, reactive oxygen species, cytokines, and lipid peroxidation. Biochem. Biophys. Res. Commun. 346, 721–726. 10.1016/j.bbrc.2006.05.159 16782068

[B156] Stone V. M. Dhayal S. Brocklehurst K. J. Lenaghan C. Sorhede Winzell M. Hammar M.. (2014). GPR120 (FFAR4) is preferentially expressed in pancreatic delta cells and regulates somatostatin secretion from murine islets of Langerhans. Diabetologia 57, 1182–1191. 10.1007/s00125-014-3213-0 24663807 PMC4018485

[B157] Su X. L. Liu Y. G. Shi M. Zhao Y. Y. Liang X. Y. Zhang L. J.. (2020). The GPR120 agonist TUG-891 inhibits the motility and phagocytosis of mouse alveolar macrophages. Biomed. Res. Int. 2020:1706168. 10.1155/2020/1706168 32149083 PMC7056993

[B158] Suckow A. T. Briscoe C. P. (2017). Key questions for translation of FFA receptors: from pharmacology to medicines. Handb. Exp. Pharmacol. 236, 101–131. 10.1007/164_2016_45 27873087

[B159] Sun P. Wang T. Zhou Y. Liu H. Jiang H. Zhu W.. (2013). DC260126: a small-molecule antagonist of GPR40 that protects against pancreatic beta-cells dysfunction in db/db mice. PLoS ONE 8:e66744. 10.1371/journal.pone.0066744 23776696 PMC3679087

[B160] Suzuki T. Igari S. Hirasawa A. Hata M. Ishiguro M. Fujieda H.. (2008). Identification of G protein-coupled receptor 120-selective agonists derived from PPARgamma agonists. J. Med. Chem. 51, 7640–7644. 10.1021/jm800970b 19007110

[B161] Takahashi H. K. Cambiaghi T. D. Luchessi A. D. Hirabara S. M. Vinolo M. A. Newsholme P.. (2012). Activation of survival and apoptotic signaling pathways in lymphocytes exposed to palmitic acid. J. Cell. Physiol. 227, 339–350. 10.1002/jcp.22740 21437903

[B162] Takashima A. Fukuda D. Tanaka K. Higashikuni Y. Hirata Y. Nishimoto S.. (2016). Combination of n-3 polyunsaturated fatty acids reduces atherogenesis in apolipoprotein E-deficient mice by inhibiting macrophage activation. Atherosclerosis 254, 142–150. 10.1016/j.atherosclerosis.2016.10.002 27744130

[B163] Tam T. H. Chan K. L. Boroumand P. Liu Z. Brozinick J. T. Bui H. H.. (2020). Nucleotides released from palmitate-activated murine macrophages attract neutrophils. J. Biol. Chem. 295, 4902–4911. 10.1074/jbc.RA119.010868 32132172 PMC7152762

[B164] Tinahones F. J. Gomez-Zumaquero J. M. Monzon A. Rojo-Martinez G. Pareja A. Morcillo S.. (2004). Dietary palmitic acid influences LDL-mediated lymphocyte proliferation differently to other mono- and polyunsaturated fatty acids in rats. Diabetes Nutr. Metab. 17, 250–258. 16295046

[B165] Torres-Hernandez A. Wang W. Nikiforov Y. Tejada K. Torres L. Kalabin A.. (2020). gammadelta T cells promote steatohepatitis by orchestrating innate and adaptive immune programming. Hepatology 71, 477–494. 10.1002/hep.30952 31529720

[B166] Unoda K. Doi Y. Nakajima H. Yamane K. Hosokawa T. Ishida S.. (2013). Eicosapentaenoic acid (EPA) induces peroxisome proliferator-activated receptors and ameliorates experimental autoimmune encephalomyelitis. J. Neuroimmunol. 256, 7–12. 10.1016/j.jneuroim.2012.12.003 23276800

[B167] Vaughan J. E. Walsh S. W. (2005). Neutrophils from pregnant women produce thromboxane and tumor necrosis factor-alpha in response to linoleic acid and oxidative stress. Am. J. Obstet. Gynecol. 193(3 Pt 1), 830–835. 10.1016/j.ajog.2005.01.057 16150282

[B168] Vazquez M. M. Gutierrez M. V. Salvatore S. R. Puiatti M. Dato V. A. Chiabrando G. A.. (2020). Nitro-oleic acid, a ligand of CD36, reduces cholesterol accumulation by modulating oxidized-LDL uptake and cholesterol efflux in RAW264.7 macrophages. Redox Biol. 36:101591. 10.1016/j.redox.2020.101591 32531545 PMC7287307

[B169] Verescakova H. Ambrozova G. Kubala L. Perecko T. Koudelka A. Vasicek O.. (2017). Nitro-oleic acid regulates growth factor-induced differentiation of bone marrow-derived macrophages. Free Radic. Biol. Med. 104, 10–19. 10.1016/j.freeradbiomed.2017.01.003 28063941 PMC5329068

[B170] Verlengia R. Gorjao R. Kanunfre C. C. Bordin S. de Lima T. M. Curi R. (2003). Effect of arachidonic acid on proliferation, cytokines production and pleiotropic genes expression in Jurkat cells–a comparison with oleic acid. Life Sci. 73, 2939–2951. 10.1016/j.lfs.2003.04.003 14519443

[B171] Verlengia R. Gorjao R. Kanunfre C. C. Bordin S. Martins De Lima T. Martins E. F.. (2004). Comparative effects of eicosapentaenoic acid and docosahexaenoic acid on proliferation, cytokine production, and pleiotropic gene expression in Jurkat cells. J. Nutr. Biochem. 15, 657–665. 10.1016/j.jnutbio.2004.04.008 15590269

[B172] Villegas-Comonfort S. Takei Y. Tsujimoto G. Hirasawa A. Garcia-Sainz J. A. (2017). Effects of arachidonic acid on FFA4 receptor: signaling, phosphorylation and internalization. Prostaglandins Leukot. Essent. Fatty Acids 117, 1–10. 10.1016/j.plefa.2017.01.013 28237082

[B173] Wang C. Liu Y. Pan Y. Jin H. (2020). Effect of GSK-137647A, the first non-carboxylic FFA4 agonist, on the osteogenic and adipogenic differentiation of bone mesenchymal stem cells in db/db mice. J. Pharm. Pharmacol. 72, 461–469. 10.1111/jphp.13217 31858612

[B174] Wang L. Ren X. Tian X. F. Cheng X. L. Zhao Y. Y. Li Q. Y.. (2019a). Protective effects of GPR120 agonist-programmed macrophages on renal interstitial fibrosis in unilateral ureteral obstruction (UUO) rats. Biomed. Pharmacother. 117:109172. 10.1016/j.biopha.2019.109172 31261028

[B175] Wang X. Chen S. He J. Chen W. Ding Y. Huang J.. (2021). Histone methyltransferases G9a mediated lipid-induced M1 macrophage polarization through negatively regulating CD36. Metab. Clin. Exp. 114:154404. 10.1016/j.metabol.2020.154404 33069810

[B176] Wang Y. Liu J. J. Dransfield P. J. Zhu L. Wang Z. Du X.. (2013). Discovery and optimization of potent GPR40 full agonists containing tricyclic spirocycles. ACS Med. Chem. Lett. 4, 551–555. 10.1021/ml300427u 24900707 PMC4027505

[B177] Wang Y. Qian Y. Fang Q. Zhong P. Li W. Wang L.. (2017). Saturated palmitic acid induces myocardial inflammatory injuries through direct binding to TLR4 accessory protein MD2. Nat. Commun. 8:13997. 10.1038/ncomms13997 28045026 PMC5216130

[B178] Wang Y. Xie T. Zhang D. Leung P. S. (2019b). GPR120 protects lipotoxicity-induced pancreatic beta-cell dysfunction through regulation of PDX1 expression and inhibition of islet inflammation. Clin. Sci. 133, 101–116. 10.1042/CS20180836 30523046

[B179] Wannick M. Bezdek S. Guillen N. Thieme M. Meshrkey F. Mousavi S.. (2018). Oral administration of the selective GPR120/FFA4 agonist compound A is not effective in alleviating tissue inflammation in mouse models of prototypical autoimmune diseases. Pharmacol. Res. Perspect. 6:e00438. 10.1002/prp2.438 30455959 PMC6223243

[B180] Wanten G. J. Janssen F. P. Naber A. H. (2002). Saturated triglycerides and fatty acids activate neutrophils depending on carbon chain-length. Eur. J. Clin. Invest. 32, 285–289. 10.1046/j.1365-2362.2002.00959.x 11952815

[B181] Watterson K. R. Hansen S. V. F. Hudson B. D. Alvarez-Curto E. Raihan S. Z. Azevedo C. M. G.. (2017). Probe-dependent negative allosteric modulators of the long-chain free fatty acid receptor FFA4. Mol. Pharmacol. 91, 630–641. 10.1124/mol.116.107821 28385906 PMC5438128

[B182] Wierenga K. A. Wee J. Gilley K. N. Rajasinghe L. D. Bates M. A. Gavrilin M. A.. (2019). Docosahexaenoic acid suppresses silica-induced inflammasome activation and IL-1 cytokine release by interfering with priming signal. Front. Immunol. 10:2130. 10.3389/fimmu.2019.02130 31616405 PMC6763728

[B183] Xiong Y. Swaminath G. Cao Q. Yang L. Guo Q. Salomonis H.. (2013). Activation of FFA1 mediates GLP-1 secretion in mice. Evidence for allosterism at FFA1. Mol. Cell Endocrinol. 369, 119–129. 10.1016/j.mce.2013.01.009 23403053

[B184] Xue B. Yang Z. Wang X. Shi H. (2012). Omega-3 polyunsaturated fatty acids antagonize macrophage inflammation via activation of AMPK/SIRT1 pathway. PLoS ONE 7:e45990. 10.1371/journal.pone.0045990 23071533 PMC3465287

[B185] Yabuki C. Komatsu H. Tsujihata Y. Maeda R. Ito R. Matsuda-Nagasumi K.. (2013). A novel antidiabetic drug, fasiglifam/TAK-875, acts as an ago-allosteric modulator of FFAR1. PLoS ONE 8:e76280. 10.1371/journal.pone.0076280 24130766 PMC3794927

[B186] Yashodhara B. M. Umakanth S. Pappachan J. M. Bhat S. K. Kamath R. Choo B. H. (2009). Omega-3 fatty acids: a comprehensive review of their role in health and disease. Postgrad. Med. J. 85, 84–90. 10.1136/pgmj.2008.073338 19329703

[B187] Yessoufou A. Ple A. Moutairou K. Hichami A. Khan N. A. (2009). Docosahexaenoic acid reduces suppressive and migratory functions of CD4+CD25+ regulatory T-cells. J. Lipid Res. 50, 2377–2388. 10.1194/jlr.M900101-JLR200 19561360 PMC2781310

[B188] Yonezawa T. Haga S. Kobayashi Y. Katoh K. Obara Y. (2008). Unsaturated fatty acids promote proliferation via ERK1/2 and Akt pathway in bovine mammary epithelial cells. Biochem. Biophys. Res. Commun. 367, 729–735. 10.1016/j.bbrc.2007.12.190 18191634

[B189] Yonezawa T. Katoh K. Obara Y. (2004). Existence of GPR40 functioning in a human breast cancer cell line, MCF-7. Biochem. Biophys. Res. Commun. 314, 805–809. 10.1016/j.bbrc.2003.12.175 14741707

[B190] Youk H. Kim M. Lee C. J. Oh J. Park S. Kang S. M.. (2021). Nlrp3, Csf3, and Edn1 in macrophage response to saturated fatty acids and modified low-density lipoprotein. Korean Circ. J. 51, 68–80. 10.4070/kcj.2020.0117 32975056 PMC7779813

[B191] Zhang L. Lu Q. Chang C. (2020). Epigenetics in health and disease. Adv. Exp. Med. Biol. 1253, 3–55. 10.1007/978-981-15-3449-2_1 32445090

[B192] Zhao Y. Zhang H. Yan A. Zhu J. Liu K. Chen D.. (2018). Grifolic acid induces mitochondrial membrane potential loss and cell death of RAW264.7 macrophages. Mol. Med. Rep. 17, 3281–3287. 10.3892/mmr.2017.8218 29257254

[B193] Zhao Y. Y. Fu H. Liang X. Y. Zhang B. L. Wei L. L. Zhu J. X.. (2019). Lipopolysaccharide inhibits GPR120 expression in macrophages via Toll-like receptor 4 and p38 MAPK activation. Cell Biol. Int. 44, 89–97. 10.1002/cbin.11204 31322778

[B194] Zhelev Z. Ivanova D. Lazarova D. Aoki I. Bakalova R. Saga T. (2016). Docosahexaenoic acid sensitizes leukemia lymphocytes to barasertib and everolimus by ROS-dependent mechanism without affecting the level of ROS and viability of normal lymphocytes. Anticancer Res. 36, 1673–1682. 10.21873/anticanres.11190 27069145

[B195] Zhou T. Wang G. Lyu Y. Wang L. Zuo S. Zou J.. (2019). Upregulation of SLAMF3 on human T cells is induced by palmitic acid through the STAT5-PI3K/Akt pathway and features the chronic inflammatory profiles of type 2 diabetes. Cell Death Dis. 10:559. 10.1038/s41419-019-1791-y 31332162 PMC6646391

